# Supplementary material for: Limiting factors for charge generation in low-offset fullerene-based organic solar cells
Source: Nat Commun. 2024 Jun 28;15:5488. doi: 10.1038/s41467-024-49432-5 (PMC11213929; doi:10.1038/s41467-024-49432-5)
Supplement: Supplementary file 1 — Supplementary Information [file 41467_2024_49432_MOESM1_ESM.pdf]

SUPPLEMENTARY INFORMATION

Limiting factors for charge generation in low-offset fullerene-based organic solar cells

Anna Jungbluth[1], Eunkyung Cho[2,†], Alberto Privitera[1,3], Kaila M. Yallum[4], Pascal Kaienburg[1], Andreas E. Lauritzen[1], Thomas Derrien[5,##], Sameer V. Kesava[1], Irfan Habib[1], Saied Md Pratik[2], Natalie Banerji[4], Jean-Luc Brédas[2], Veaceslav Coropceanu[2], Moritz Riede[1,]*

1. Department of Physics, The University of Oxford, Oxford, Oxfordshire OX13PJ, UK
2. Department of Chemistry and Biochemistry, The University of Arizona, Tucson, Arizona 85721-0088, USA
3. Department of Industrial Engineering and INSTM Research Unit, University of Florence, 50139, Firenze, Italy
4. Department of Chemistry, Biochemistry and Pharmaceutical Sciences, University of Bern, 3012 Bern, Switzerland.
5. Diamond Light Source, Didcot, Oxfordshire OX11 0DE, UK

† Current Address: Division of Energy Technology, DGIST, Daegu, 42988, Republic of Korea

# Current Address: Living Systems Institute, University of Exeter, Exeter EX4 4QD, UK

* Corresponding Author; moritz.riede@physics.ox.ac.uk

SUPPLEMENTARY FIGURES

Donor	100 %	5 %
ZnPc	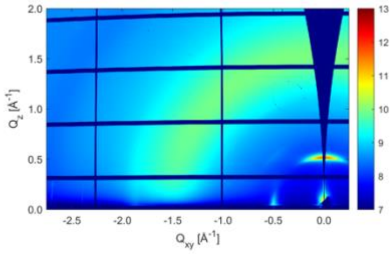	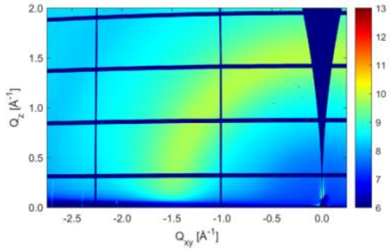
F_4ZnPc	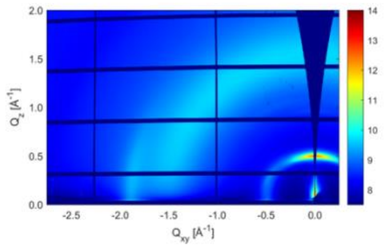	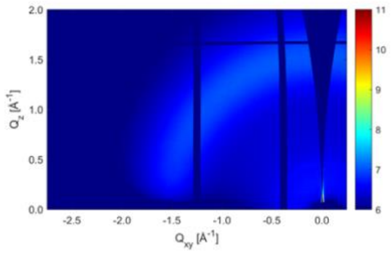
F_8ZnPc	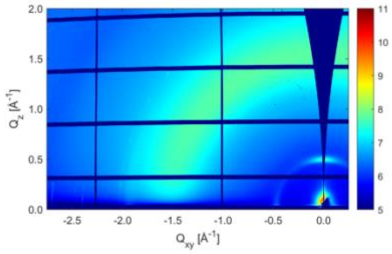	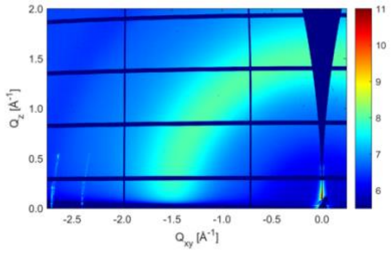
$F_{16}ZnPc$	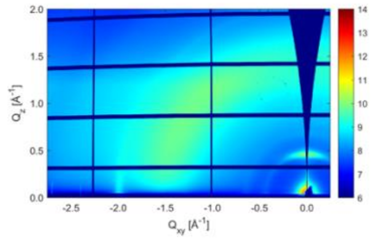	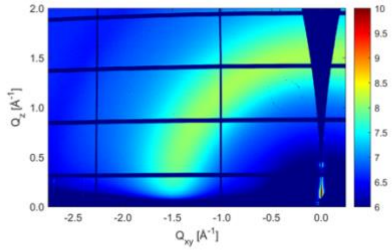

Figure S1. 2D Grazing incidence wide-angle X-ray (GIWAXS) reciprocal space maps of 50 nm thin films on quartz. The first column shows scattering of the neat F_xZnPc films, the second column shows scattering of the dilute blends. The primary out-of-plane peak is indexed as the (200) peak and the primary in-plane peak is indexed as the (010) peak.

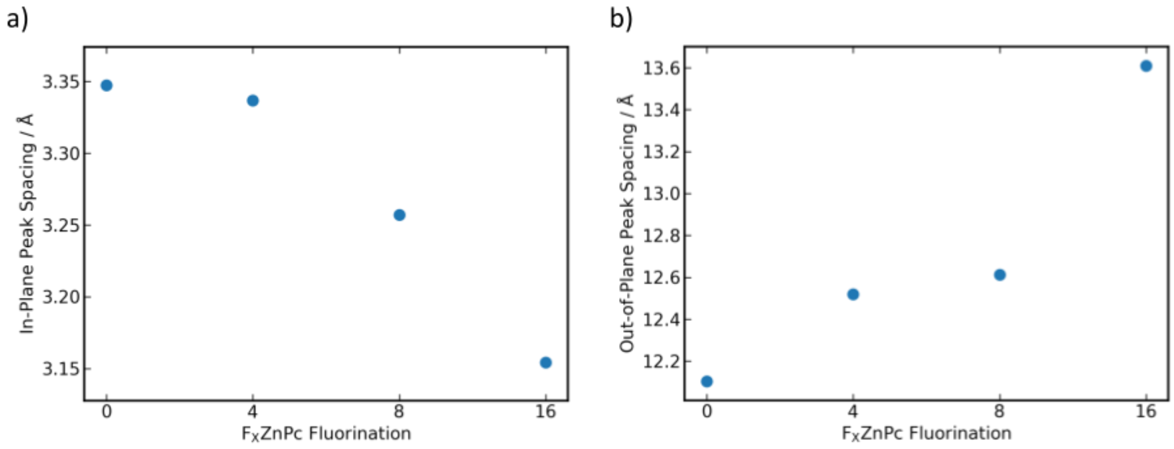

Figure S2. Real spacing as a function of donor fluorination. The real spacing of the a) in-plane (010) and b) out-of-place (200) peaks were determined via analysis of the grazing incidence wide-angle X-ray (GIWAXS) measurements of neat F_xZnPc films on quartz.

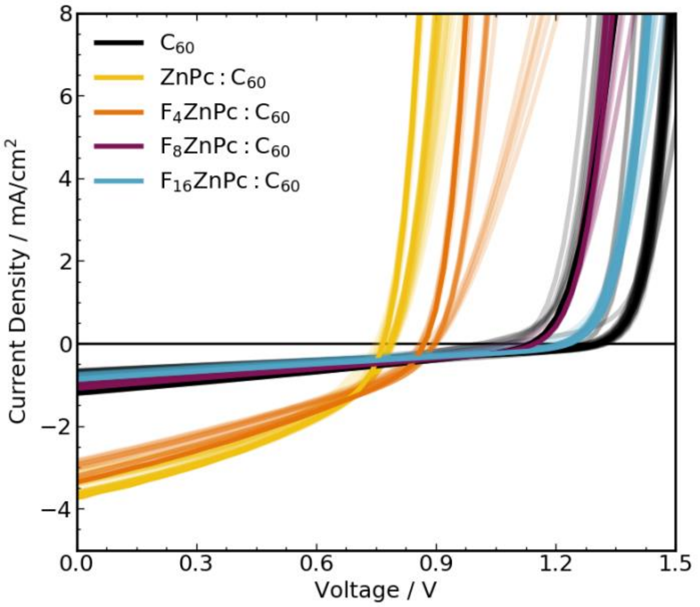

Figure S3. Current density-voltage (J – V) characteristics measured for multiple devices based on dilute F_xZnP:C_{60} blends or neat C_{60}. All measurements were performed under simulated 1 Sun illumination. The corresponding performance trends are shown in Table S1. The spread of measured J – V characteristics is largest for the C_{60} reference devices. This is because the neat C_{60} devices were fabricated across two years and likely encompass deviations across sample fabrication conditions (e.g., cleanliness of the evaporation chamber).

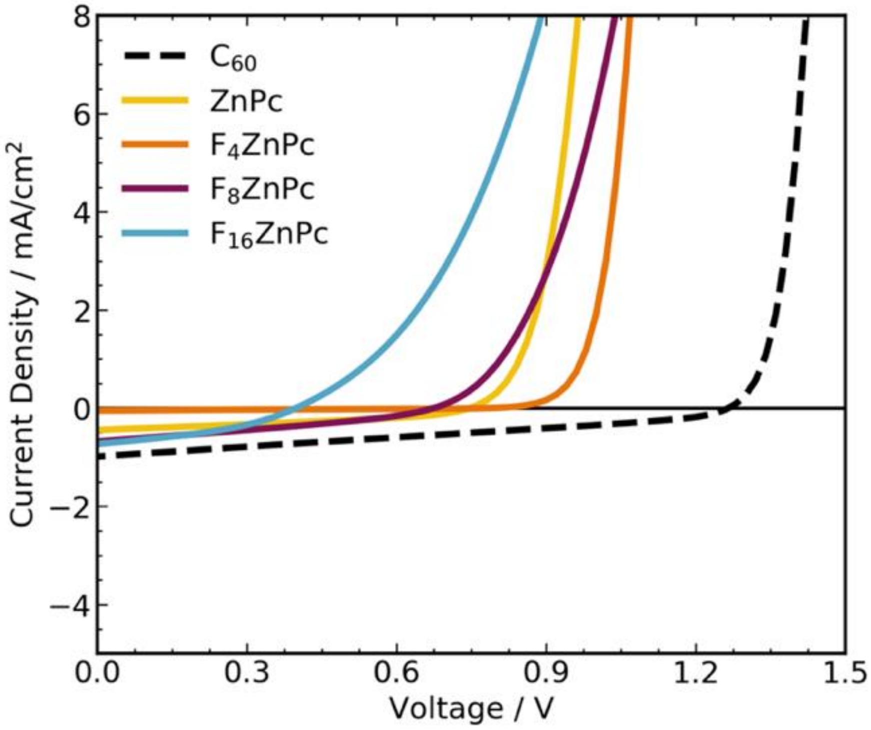

Figure S4. Current density-voltage characteristics measured for neat reference devices. All measurements were performed under simulated 1 Sun illumination for the best performing (highest open-circuit voltage) neat F_xZnPc solar cells.

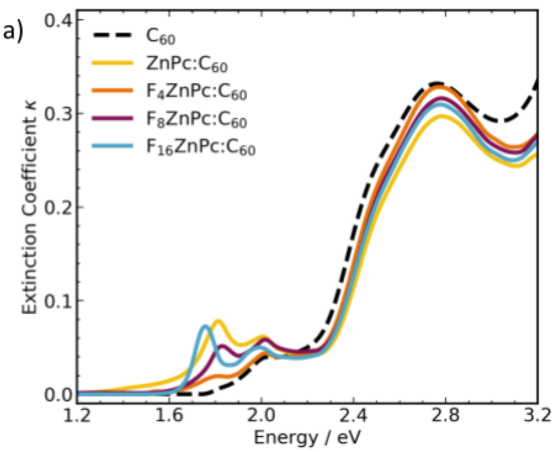

Figure S5. Extinction coefficient (κ) measured via spectroscopic ellipsometry. The measurements were performed for thin films on quartz of a) dilute $F_xZnPc:C_{60}$ and b) neat ZnPc, F_8ZnPc, and C_{60}. The absorbance of the dilute blends strongly follows that of neat C_{60}, with only minor changes between 1.6 – 2.2 eV caused by the characteristic absorption doublet of F_xZnPc.[2,3] Compared to the absorption profile of ZnPc, the slight red shift (blue shift) of the absorption peaks of F_4ZnPc and $F_{16}ZnPc$ (F_8ZnPc) is ascribed to changes in the transition dipole alignment and intermolecular coupling upon fluorination.[2]

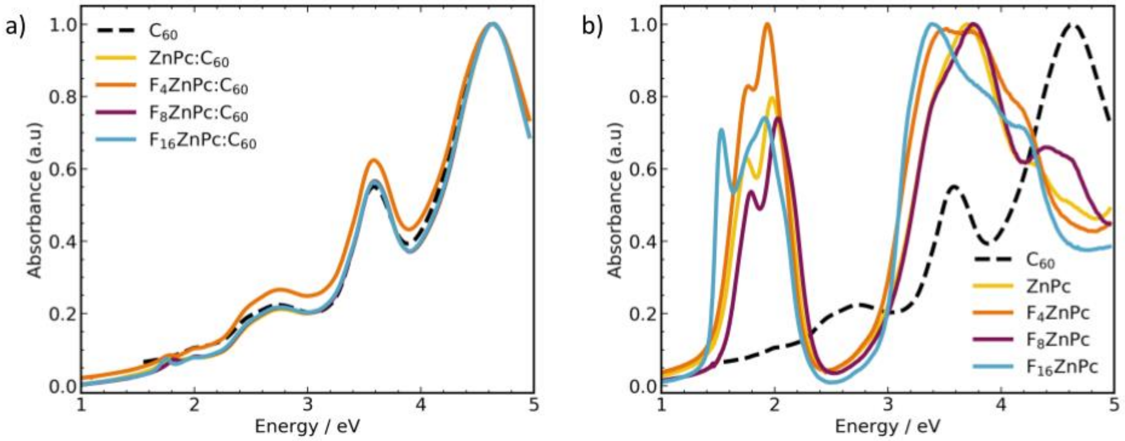

Figure S6. Normalized absorbance measured via ultraviolet-visible spectroscopy. The measurements were performed on thin films of a) dilute $F_xZnPc:C_{60}$ and b) the neat films on quartz.

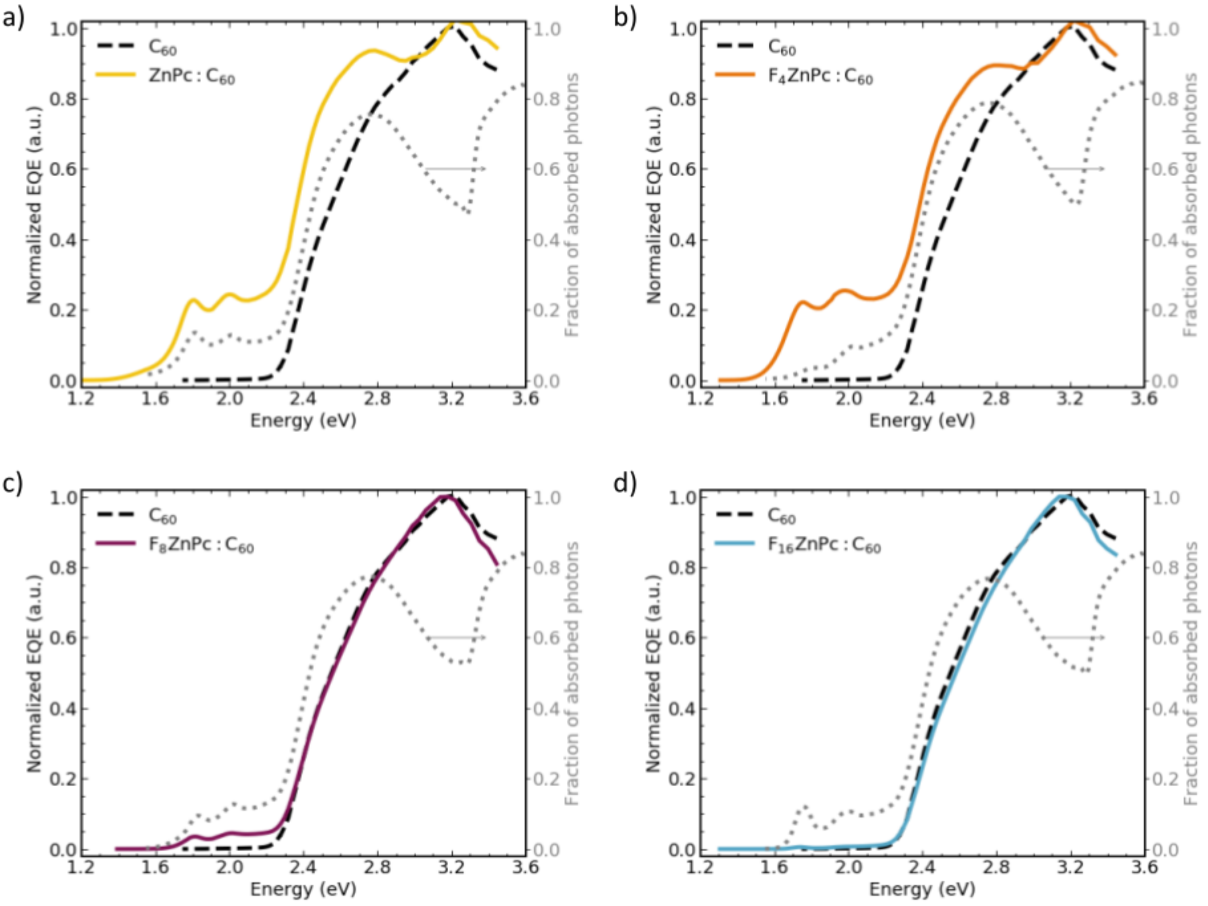

Figure S7. Comparison of photon absorption (right axis) and charge generation (left axis). The measurements were performed for dilute donor blends of a) ZnPc, b) F_4ZnPc, c) F_8ZnPc, and d) F_{16}ZnPc and C_{60}. The complex refractive index of the blends was measured via spectroscopic ellipsometry, and the fraction of absorbed photons calculated using the transfer matrix approach.[4] This is compared to the external quantum efficiency (EQE) at room temperature of full devices with the respective blends as active layers. All material systems show photon absorption between 1.5 – 2.2 eV, but only the ZnPc and F_4ZnPc blends show efficient free charge generation in this spectral region.

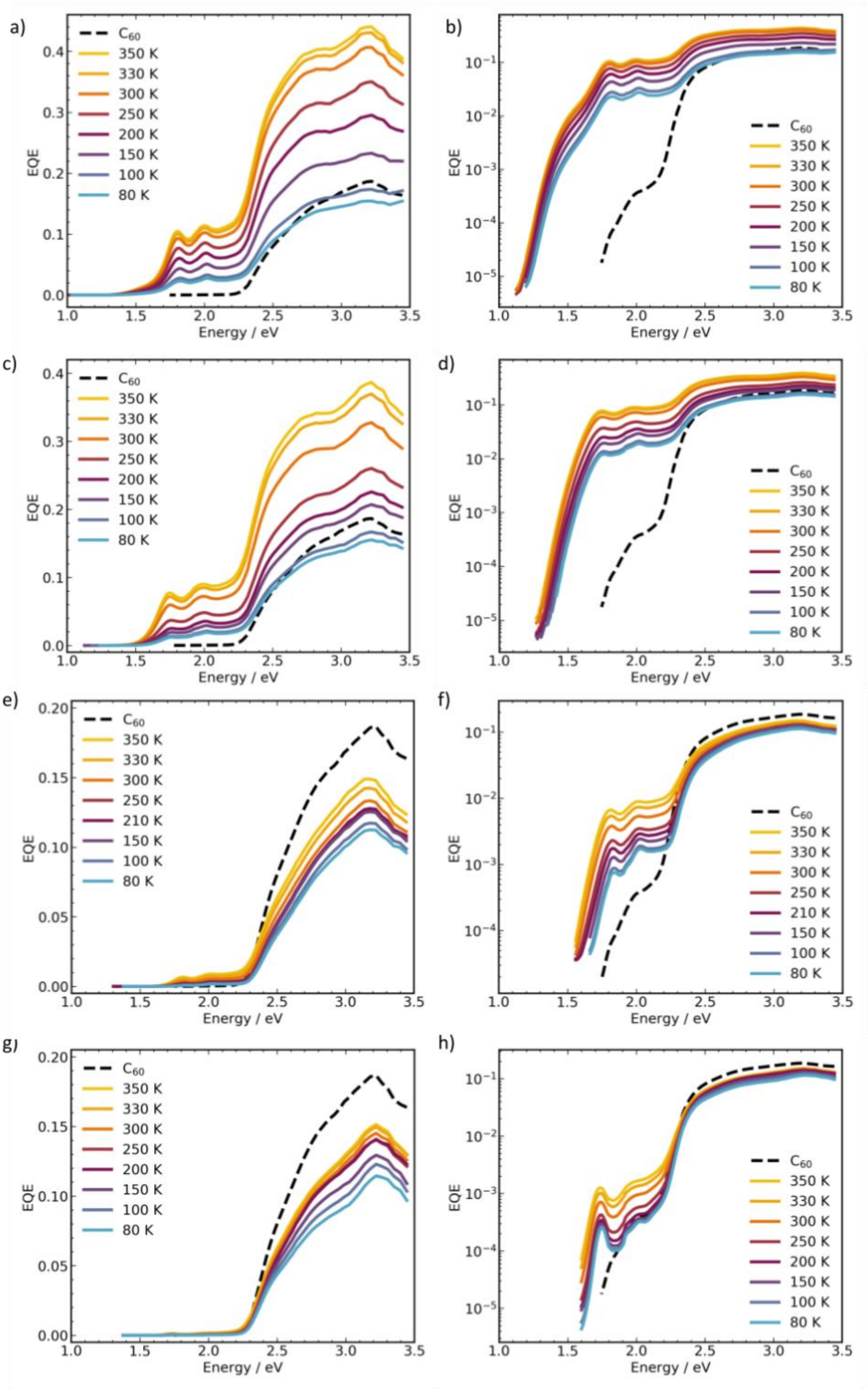

Figure S8. Temperature-dependent external quantum efficiency (EQE) spectra. The EQE was measured for dilute blends of a) - b) ZnPc, c) - d) F_4ZnPc, e) - f) F_8ZnPc, g) - h) $F_{16}ZnPc$ and C_{60}.

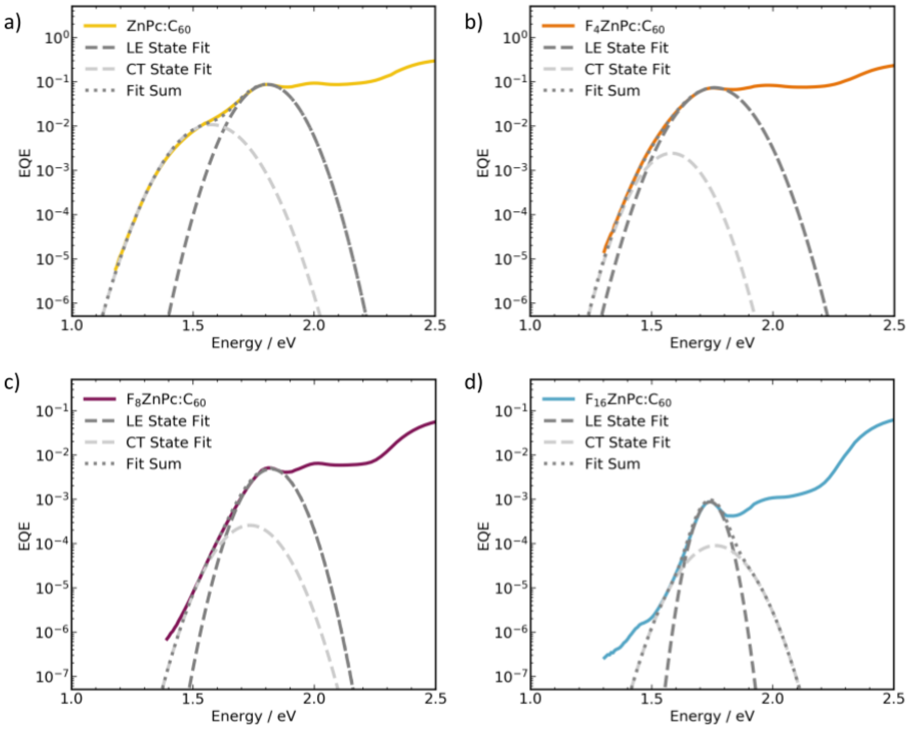

Figure S9. Fitting of room temperature external quantum efficiency (EQE) spectra. The EQE measurements were performed for dilute donor blends of a) ZnPc, b) F_4ZnPc, c) F_8ZnPc, and d) F_{16}ZnPc in C_{60}. The light and dark grey dashed lines show the fits of charge transfer (CT) and local excitation (LE) singlet states, respectively. The dotted lines indicate the sum of the fits. Both the LE and CT states were fit using classical Marcus theory,[5,6] and following the methodology outlined in [7–9].

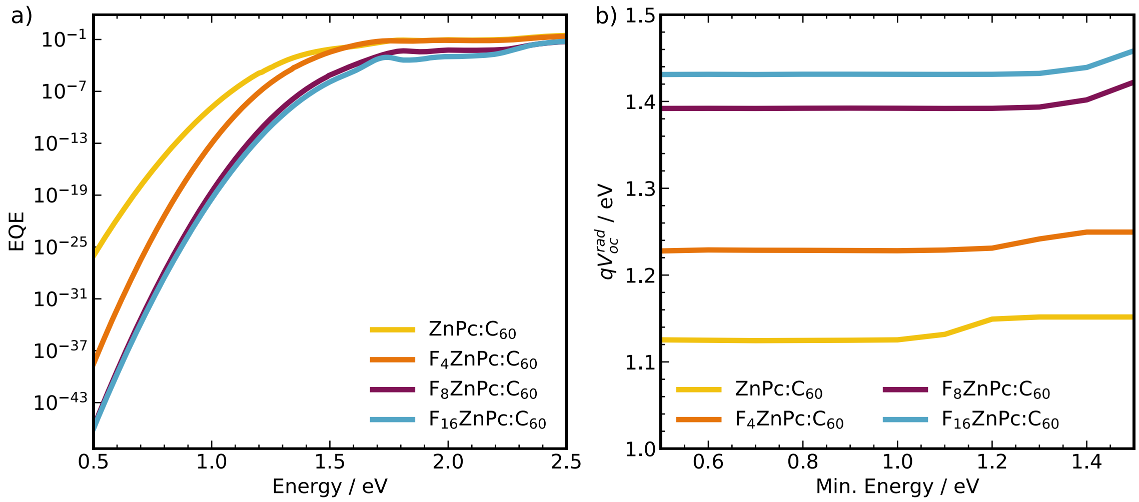

Figure S10. Determination of the radiative energy limit of the open circuit voltage (qV_{oc}^{rad}). To ensure that qV_{oc}^{rad} saturates, all external quantum efficiency (EQE) spectra are extended using the gaussian functions determined via Marcus theory fitting of the charge transfer state. The resulting EQE spectra, extended to a minimum energy of 0.5 eV, are shown in a). The dependence of qV_{oc}^{rad} on the minimum energy is shown in b).

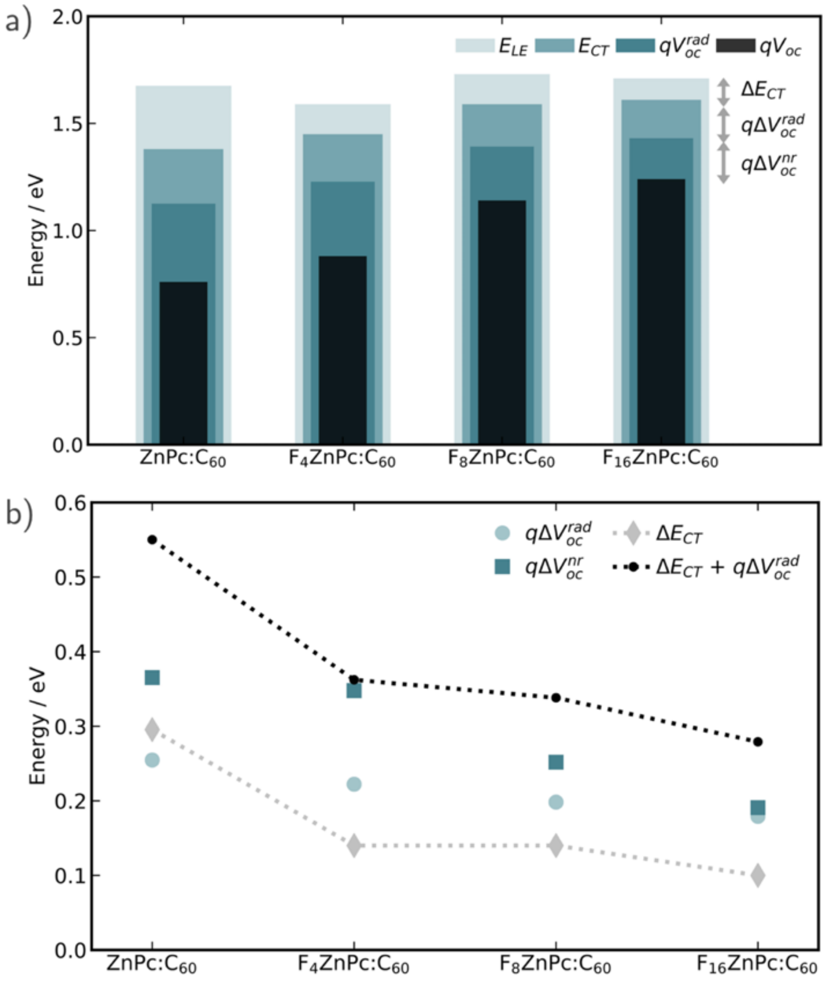

0

Figure S11. Energy levels and energy losses derived for our studied material systems. a) The energy of the local excitation singlet (E_{LE}) and charge transfer state (E_{CT}) were calculated via Marcus theory fitting of external quantum efficiency (EQE) spectra. The radiative energy limit of the open circuit voltage (qV_{oc}^{rad}) was determined via integration of the EQE, and the open circuit voltage (V_{oc}) was determined through current density – voltage measurements. b) From these energy levels, the driving force for charge transfer (ΔE_{CT}), and radiative ($q\Delta V_{oc}^{rad}$) and non-radiative ($q\Delta V_{oc}^{nr}$) energy losses were calculated as described above. We note that the energy levels and energy losses were only calculated for the best performing samples, and no in-depth uncertainty quantification was performed.

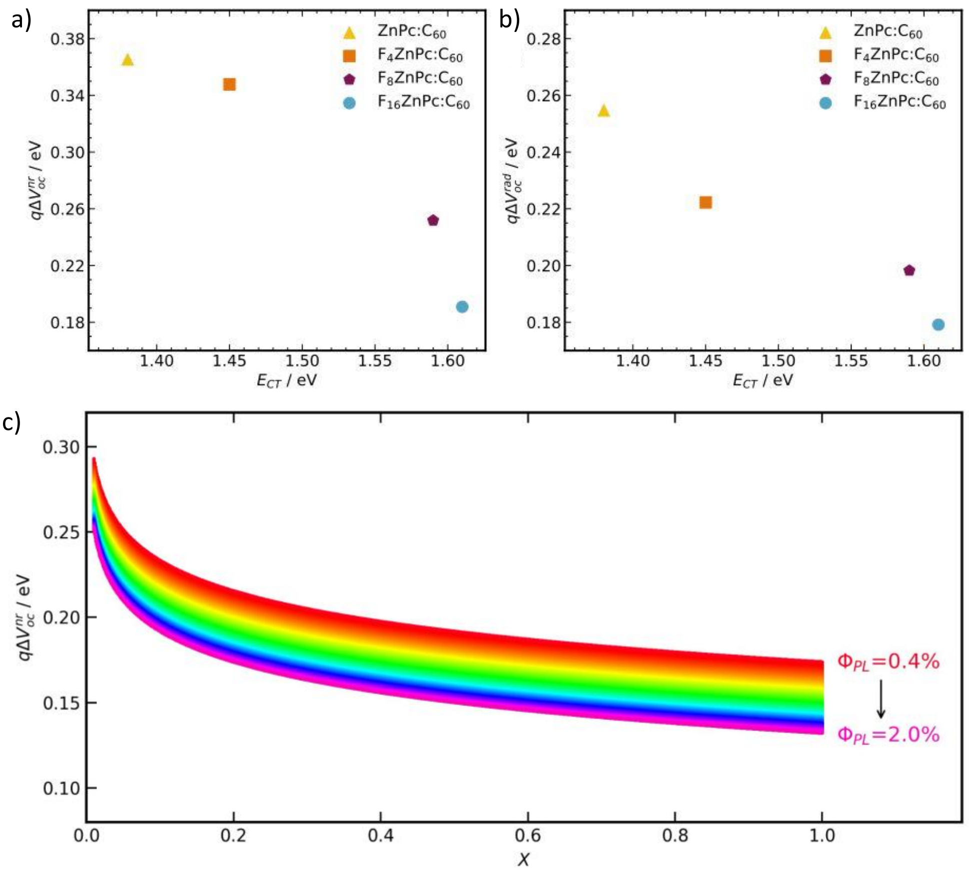

Figure S12. Dependence of energy losses on charge transfer state energies (E_{CT}; top) and radiative recombination events (bottom). a) Non-radiative energy losses ($q\Delta V_{oc}^{nr}$) and b) radiative energy losses ($q\Delta V_{oc}^{rad}$) decrease with increasing E_{CT}. c) $q\Delta V_{oc}^{nr}$ also depends on the fraction of radiative recombination events (χ) and photoluminescence quantum efficiency (ϕ_{PL}), modelled after equations 1 and 2 in Gillett et al.[12] We gradually increased ϕ_{PL} from 0.4% to 2.0%, which is reasonable for organic solar cells. We also assume a photon out-coupling efficiency of 0.3, a charge balance factor of 1, and a temperature of 300 K.[12] When χ increases, $q\Delta V_{oc}^{nr}$ decreases. Similarly, when ϕ_{PL} increases, $q\Delta V_{oc}^{nr}$ is reduced. The latter can be achieved, for instance, by increasing E_{CT} and reducing non-radiative coupling between the CT and ground state (according to the energy gap law[13]). As a result, the total $q\Delta V_{oc}^{nr}$ measured for different material systems arises from the interplay between these different factors.

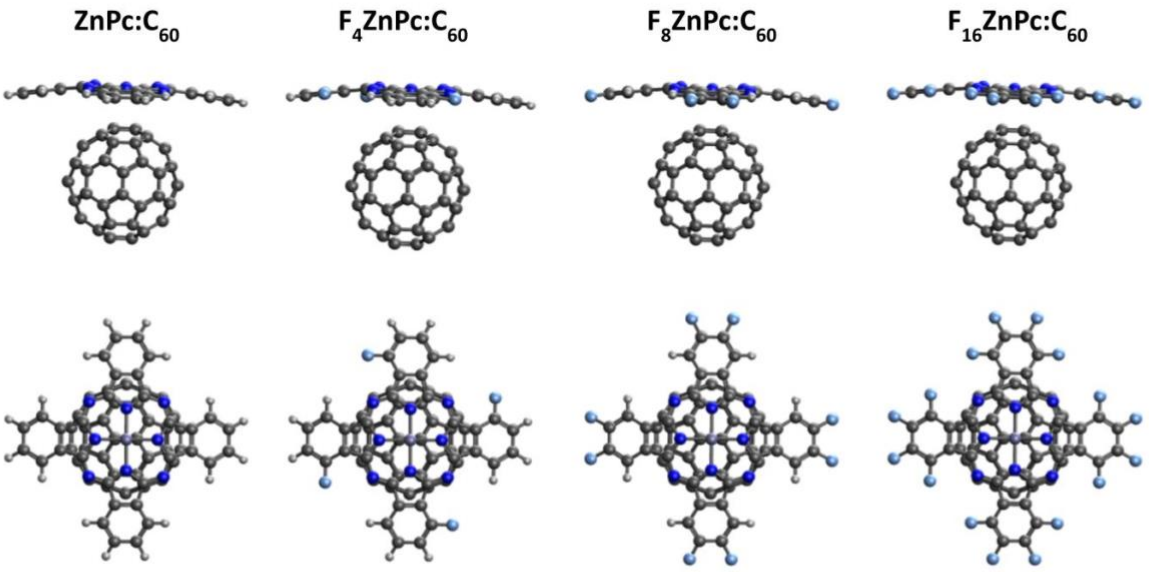

Figure S13. Optimized structures of isolated $F_xZnPc:C_{60}$ complexes.

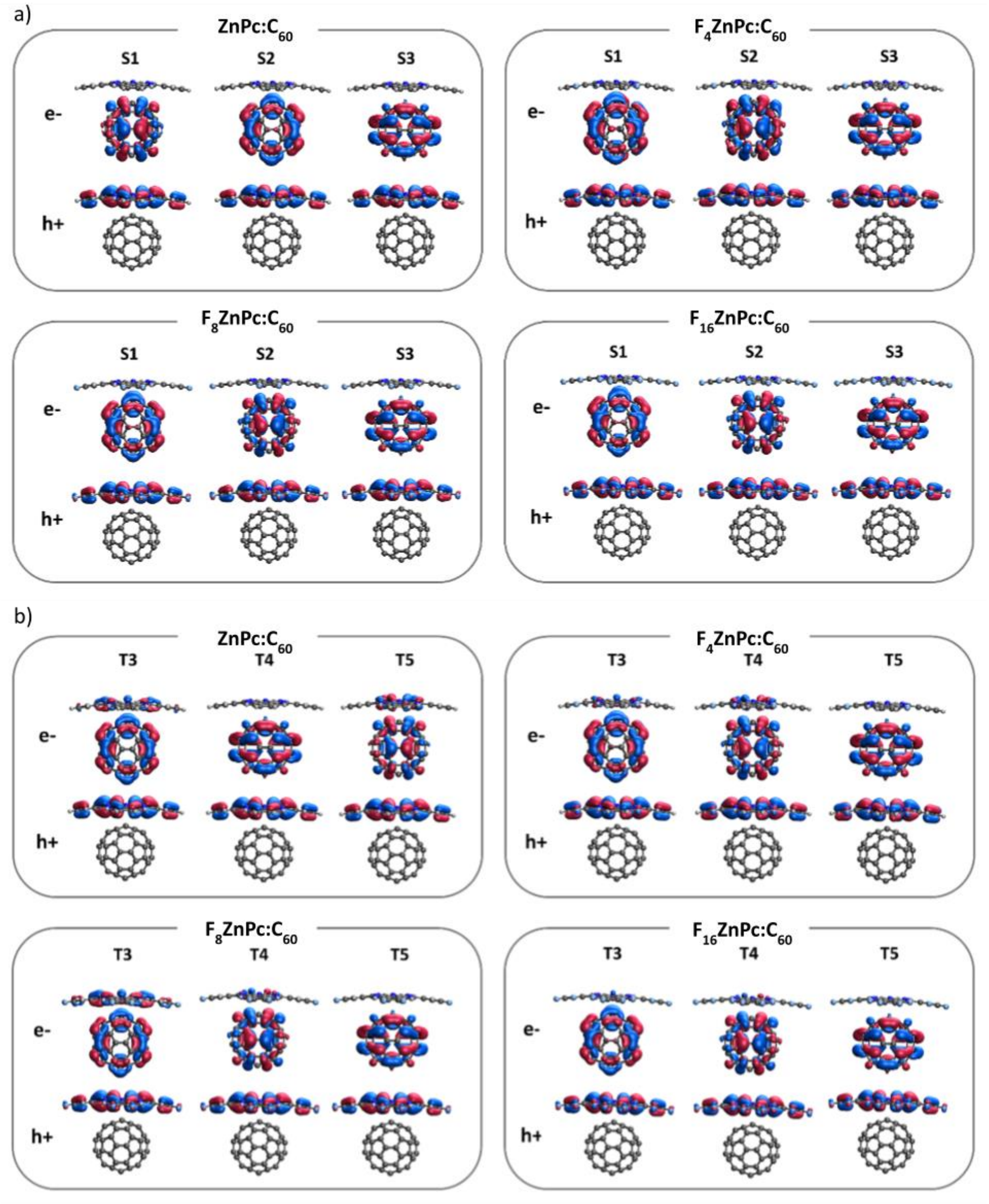

Figure S14. Electron and hole natural transition orbitals (NTOs). The NTOs of the three lowest a) singlet and b) triplet charge transfer (CT) states of the $F_xZnPc:C_{60}$ complexes were calculated by considering a dielectric medium with ε=3.

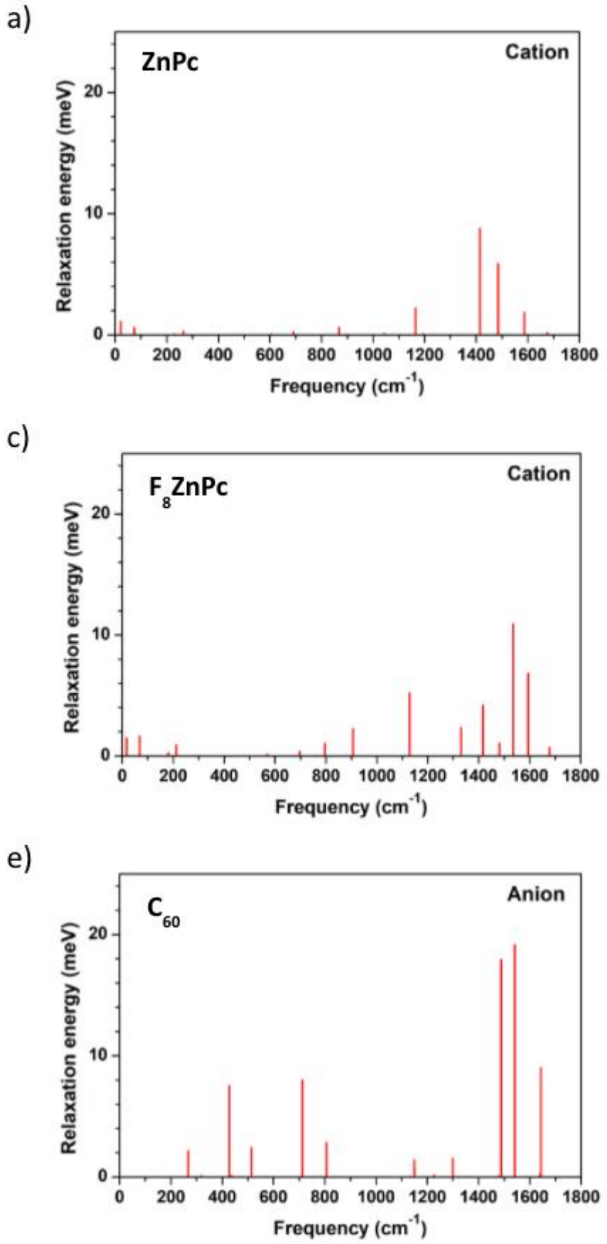

Figure S15. Decomposition of the relaxation energies of isolated a) ZnPc, b) F_4ZnPc, c) F_8ZnPc, d) F_{16}ZnPc, and e) C_{60} molecules into normal mode contributions. In all cases, the high-frequency vibrations (1400-1600 cm^{-1}) dominate.

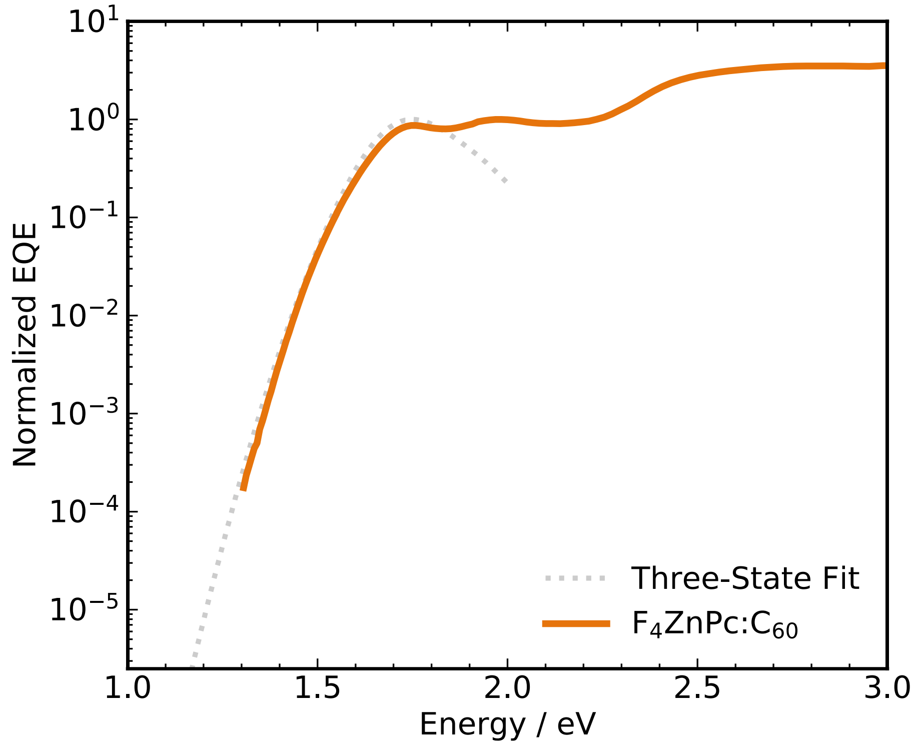

Figure S16. Three-state vibronic model fitting of $F_4ZnPc:C_{60}$. All corresponding fit values are included in Table S12.

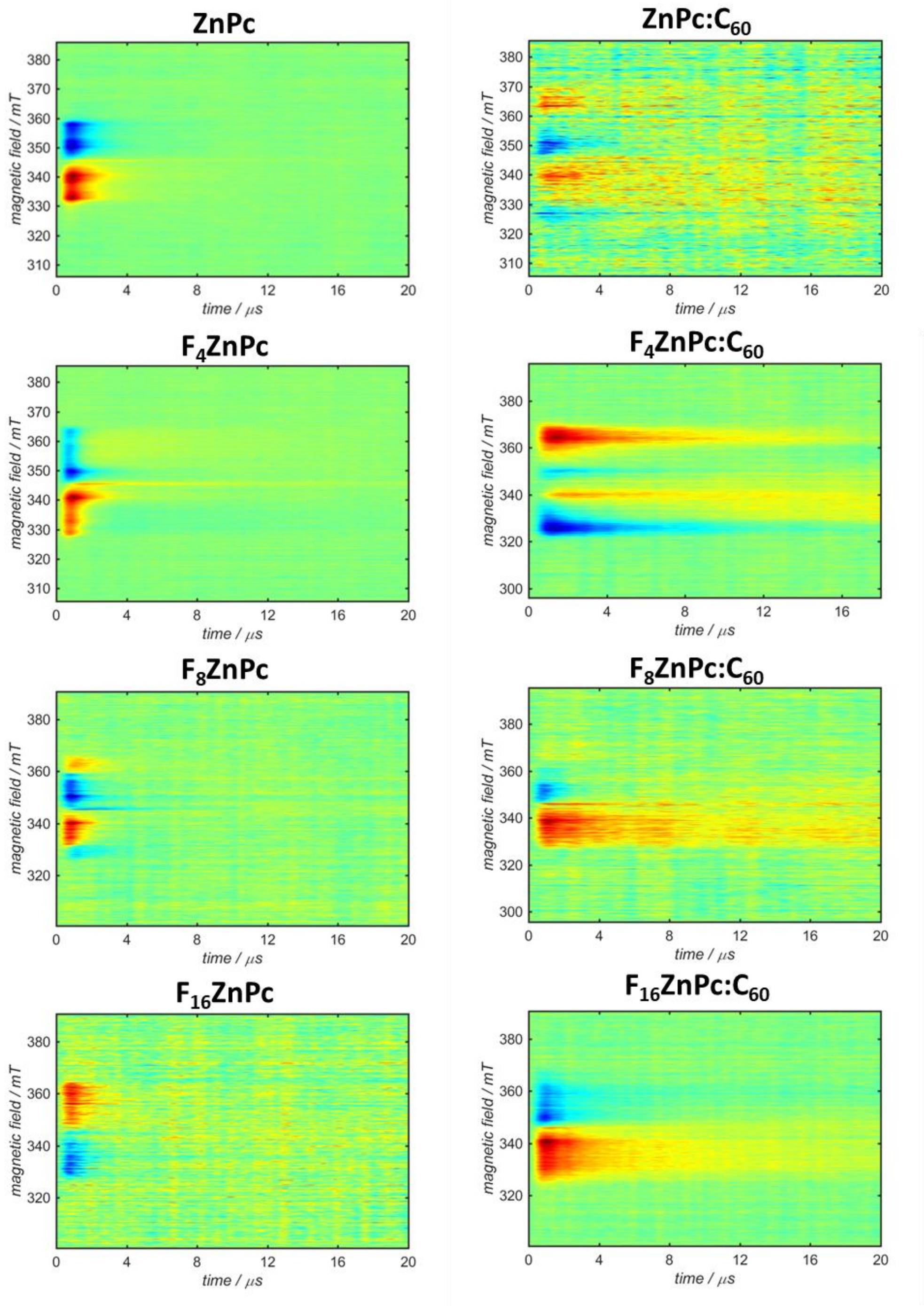

Figure S17. Two-dimensional time-resolved electron paramagnetic resonance (trEPR) spectra of all neat (first column) and dilute donor (second column) films studied in this work. The trEPR spectra were acquired at 80 K and after excitation at 532 nm. In this figure, emission is highlighted in blue, and absorption is highlighted in red.

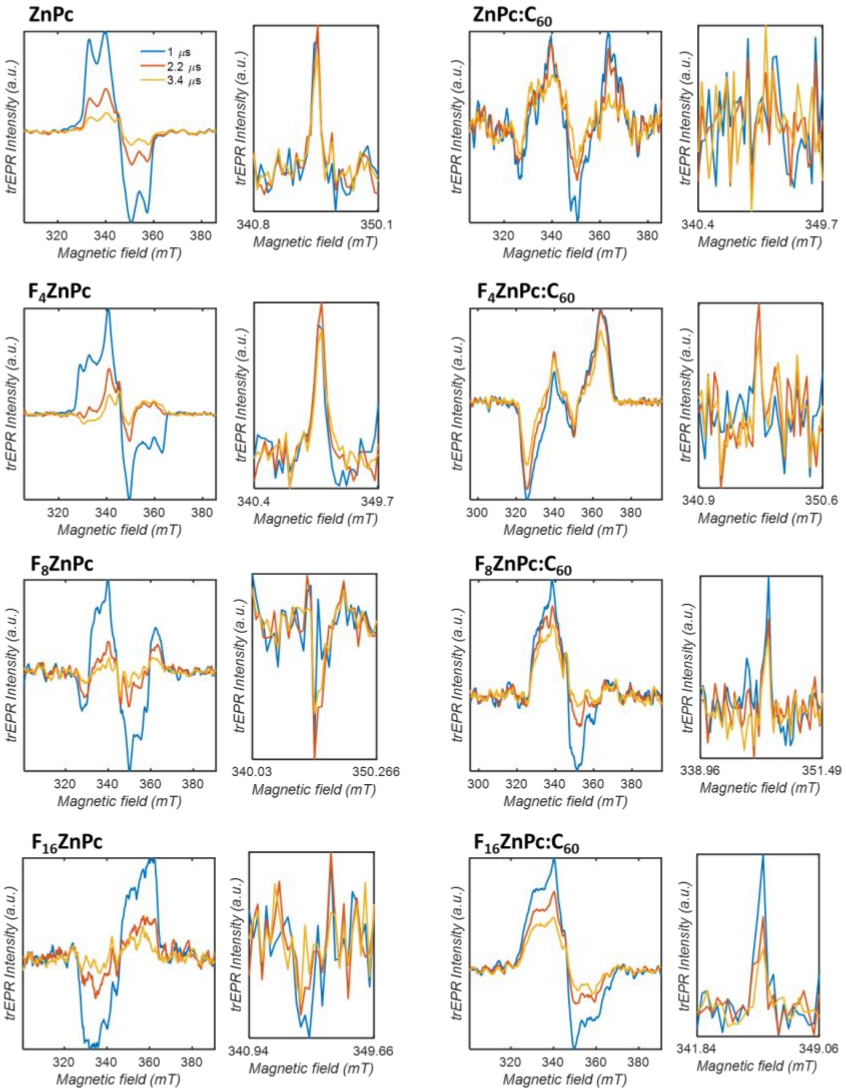

Figure S18. One-dimensional time-resolved electron paramagnetic resonance (trEPR) spectra of all neat (first and second column) and dilute donor (third and fourth column) films. The trEPR spectra were acquired at 80 K and after excitation at 532 nm. Absorption (*a*) is up, and emission (*e*) is down. For each sample, three representative time delays after the initial laser pulse are reported. Legend: blue = 1μs, red = 2.2 μs, orange = 3.4 μs. For each sample, the left figure (first and third column) shows the full magnetic field sweep to highlight the time evolution of the triplet states, while the right figure (second and fourth column) shows a narrow sweep magnification of the radical pair signal.

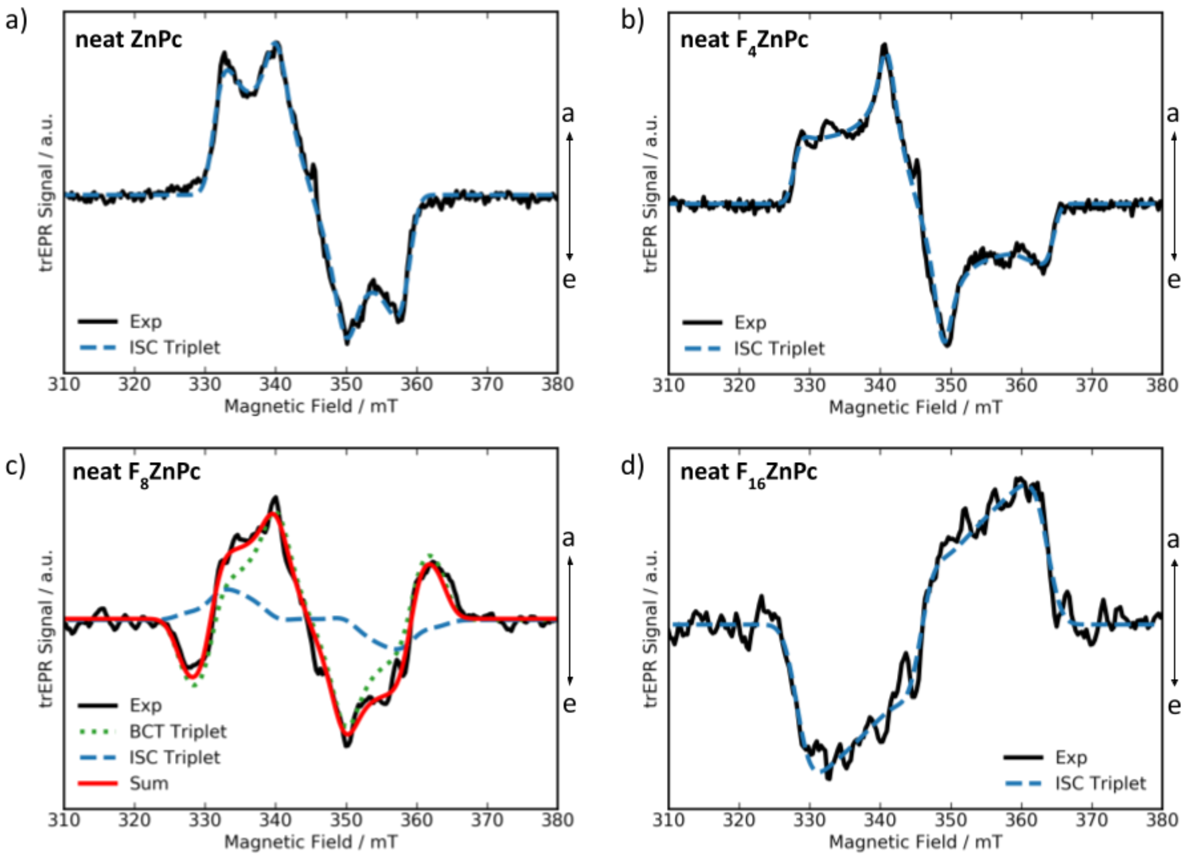

Figure S19. Time-resolved electron paramagnetic resonance (trEPR) spectra and spectral fits. Measurements were performed for neat a) ZnPc, b) F_4ZnPc, c) F_8ZnPc, and d) F_{16}ZnPc films and at 80 K. Black line: trEPR spectra of the dilute blends recorded at 1 µs (integration window 0.8-1.2 µs) after a 532 nm laser pulse. Absorption (*a*) is up, and emission (*e*) is down. All spectra show two main species: (1) a narrow signal in neat absorption or emission centered around 345 mT which can be attributed to photogenerated charges and (2) a broad signal ranging from about 320 to 370 mT which can be attributed to triplet states. Red line: best-fit spectral simulations of donor triplets. In the case of neat ZnPc, F_4ZnPc, and F_{16}ZnPc, triplet states are populated through inter-system crossing (ISC) while neat F_8ZnPc shows both an inter-system crossing and a back electron transfer (BET) contribution. The values of the best-fit simulations are reported in Table S13.

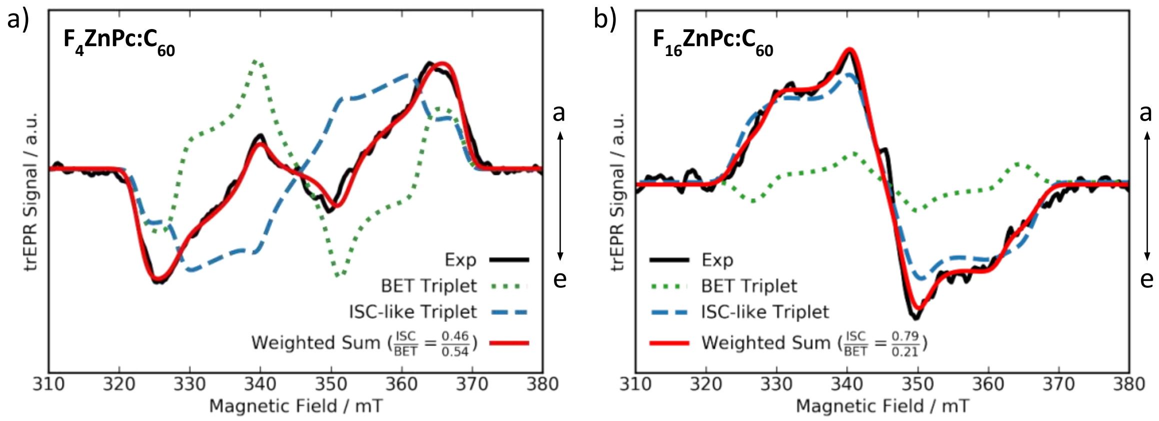

Figure S20. Time-resolved electron paramagnetic resonance (trEPR) spectra and spectral fits. Measurements were performed for dilute a) F_4ZnPc, and b) F_{16}ZnPc blends and at 80 K. Black line: trEPR spectra of the dilute blends recorded at 1 μs (integration window 0.8-1.2 μs) after a 532 nm laser pulse. Absorption (*a*) is up and emission (*e*) is down. The narrow signals centered around 345 mT correspond to photogenerated charges, while the broad peaks between 320 – 370 mT correspond to triplet states. Red line: best-fit spectral simulations of donor triplets obtained as the sum of two contributions: (1) intersystem crossing (ISC)-like contribution (blue dashed line) and (2) geminate back electron transfer (BET) to a low-lying T_1 triplet state (dotted green line). The values of the best-fit simulations are reported in the main paper. We note that for the dilute F_{16}ZnPc blend, a decent fit was also achieved without considering BET (Figure S21).

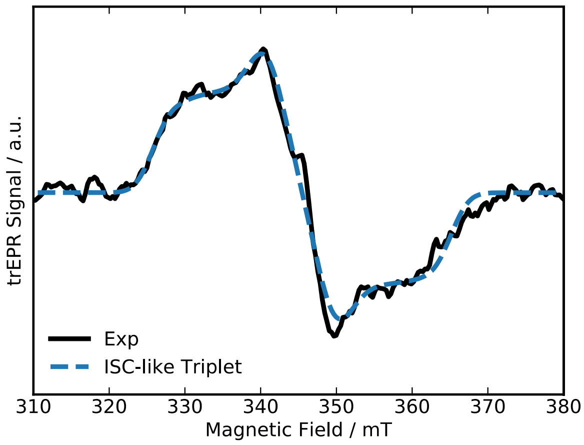

Figure S21. Time-resolved electron paramagnetic resonance (trEPR) spectrum and spectral fit. The measurement was performed at 80 K on the dilute F_{16}ZnPc blend (black line), considering only an intersystem crossing (ISC)-like contribution (blue dashed line) during fitting.

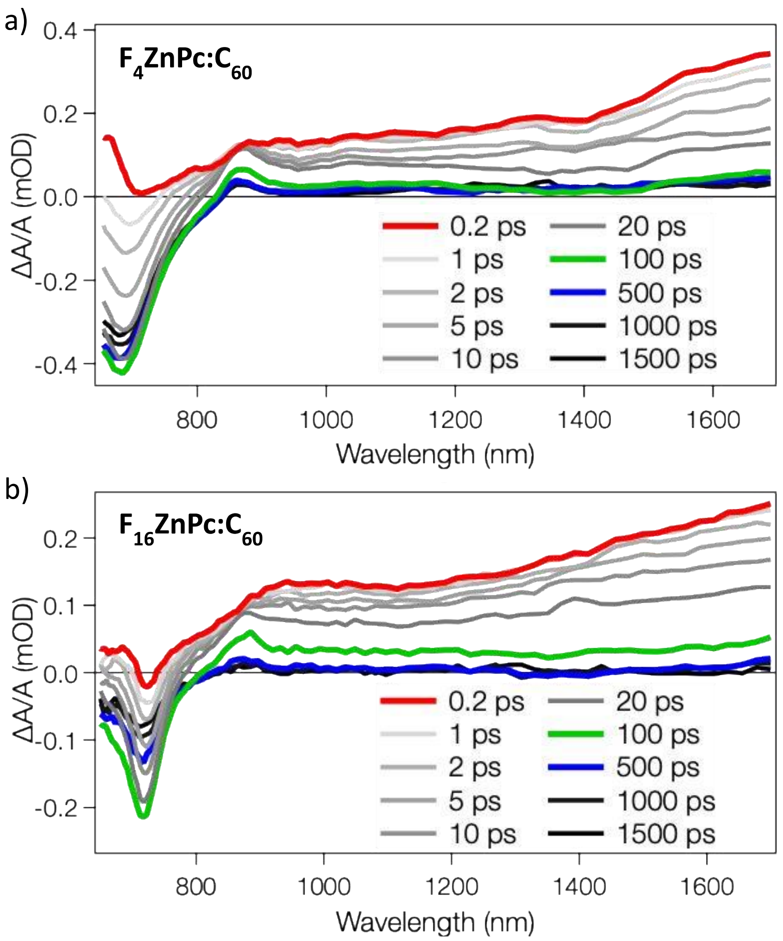

Figure S22. Transient absorption (TA) spectra of fluorinated blends. The measurements were performed for 0.2 – 1500 ps after excitation for dilute a) $F_4ZnPc:C_{60}$, and b) $F_{16}ZnPc:C_{60}$. All samples show an increase in the ground-state bleach (GSB) of the donor between 0.2 and 50 ps. As the initial excitation at 530 nm mostly excites C_{60} (see Figure S6), this increase in GSB of the donor is indicative of electron and/or energy transfer from the excited C_{60} to the donor.

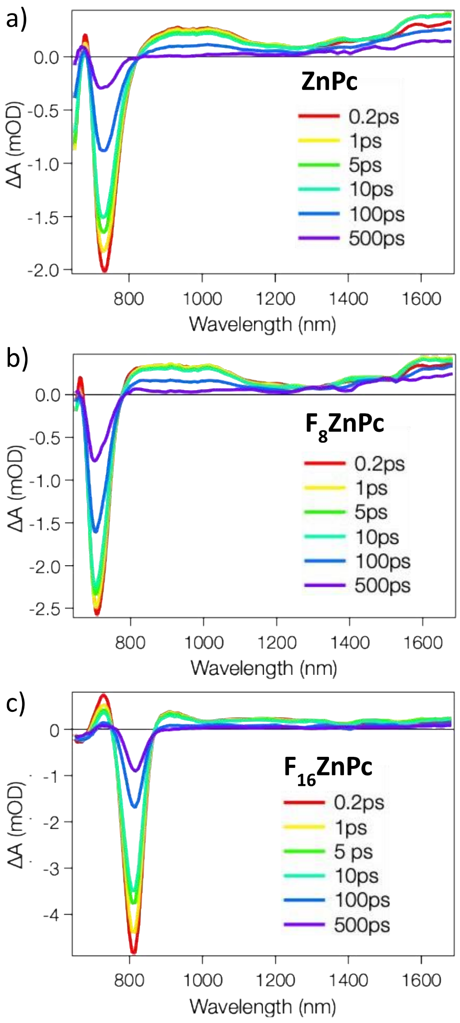

Figure S23. Transient absorption (TA) spectra of the neat donor films. The measurements were performed after excitation for neat a) ZnPc, b) F_8ZnPc and c) F_{16}ZnPc. The early spectra mainly show singlet excitons, while the late spectra consist of charges and/or triplet excitons. Spectral signatures cannot be directly compared to the dilute blends, since the neat films show donor aggregation.

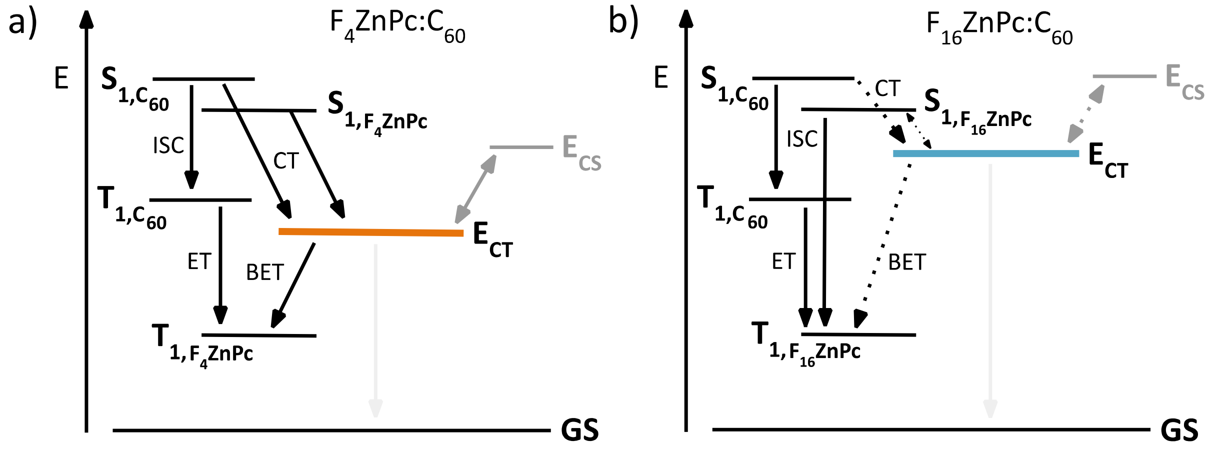

Figure S24. Schematic representations of computationally and experimentally derived energy levels and dominant transition pathways. The charge transfer (CT), intersystem crossing (ISC), back electron transfer (BET), and energy transfer (ET) processes are shown for dilute a) F_4ZnPc, and b) $F_{16}ZnPc$ blends with C_{60}. The dotted lines signal pathways that we observe with lower probability. The represented CT state is comprised of nearly degenerate singlet and triplet CT states. Although not shown in the diagram, all excited states can directly recombine to the ground state (GS).

SUPPLEMENTARY TABLES

Table S1. Device performance of dilute $F_xZnPc:C_{60}$ and neat C_{60} devices. All measurements were performed under simulated 1 Sun illumination. The mean and standard deviation of the open-circuit voltage (V_{OC}), short-circuit current (J_{SC}), fill factor (FF), and power conversion efficiency (PCE) were determined for > 10 devices for all material systems, with the exact number of devices shown in the last column. The data were not mismatch corrected. However, due to the strong similarities in the absorption profile (see Figure S5 and Figure S6), mismatch correction would result in similar scaling for all samples and would preserve the observed performance trends.

	V_{oc} [V]	FF [%]	J_{sc} [mA cm^{-2}]	PCE [%]	#
$ZnPc:C_{60}$	0.78 ± 0.02	40 ± 1	3.6 ± 0.2	1.10 ± 0.06	19
$F_4ZnPc:C_{60}$	0.89 ± 0.02	33 ± 1	3.1 ± 0.2	0.92 ± 0.06	13
$F_8ZnPc:C_{60}$	1.14 ± 0.02	30 ± 1	1.0 ± 0.1	0.35 ± 0.02	12
$F_{16}ZnPc:C_{60}$	1.21 ± 0.02	29 ± 1	0.8 ± 0.1	0.28 ± 0.02	15
C_{60} Reference	1.25 ± 0.09	30 ± 1	0.9 ± 0.2	0.33 ± 0.04	30

Table S2. Results of Marcus theory fitting of local excitation (LE) singlet and charge transfer (CT) states of the dilute blends. Uncertainties are given as the errors on the fit, rather than deviations across samples.

	$ZnPc:C_{60}$	$F_4ZnPc:C_{60}$	$F_8ZnPc:C_{60}$	$F_{16}ZnPc:C_{60}$
E_{LE} (eV)	1.68 ± 0.01	1.59 ± 0.01	1.73 ± 0.01	1.71 ± 0.01
E_{CT} (eV)	1.38 ± 0.01	1.45 ± 0.01	1.59 ± 0.01	1.61 ± 0.01
λ_{CT} (eV)	0.199 ± 0.01	0.136 ± 0.03	0.151 ± 0.01	0.159 ± 0.01

Table S3. Overview of energy losses. The driving force for charge transfer (ΔE_{CT}), radiative ($q\Delta V_{oc}^{rad}$) and non-radiative ($q\Delta V_{oc}^{nr}$) energy losses were calculated for all dilute donor blends.

	$ZnPc:C_{60}$	$F_4ZnPc:C_{60}$	$F_8ZnPc:C_{60}$	$F_{16}ZnPc:C_{60}$
ΔE_{CT} (eV)	0.300	0.140	0.140	0.100
$q\Delta V_{oc}^{rad}$ (eV)	0.250	0.220	0.200	0.180
$q\Delta V_{oc}^{nr}$ (eV)	0.370	0.350	0.250	0.190

Table S4. Calculated singlet and triplet energies of the ZnPc:C_{60} complex as a function of dielectric constant. The charge transfer (CT) and local excitation (LE) singlet ZnPc states are shown in red and blue, respectively.

State	[eV]	ε=2	ε=3	ε=3.5	ε=4
S1	CT	1.46	1.36	1.32	1.30
S2	CT	1.46	1.36	1.32	1.30
S3	CT	1.53	1.41	1.37	1.34
S4	LE-C_{60}	2.22	2.15	2.13	2.12
S5	LE-C_{60}	2.23	2.16	2.14	2.13
S6	LE-C_{60}	2.23	2.17	2.15	2.14
S7	LE-C_{60}	2.23	2.17	2.15	2.14
S8	LE-C_{60}	2.24	2.18	2.16	2.15
S9	LE-C_{60}	2.25	2.19	2.17	2.15
S10	LE-C_{60}	2.25	2.19	2.17	2.16
S11	LE-C_{60}	2.26	2.20	2.18	2.17
S12	LE-C_{60}	2.26	2.21	2.20	2.18
S13	LE-C_{60}	2.27	2.21	2.19	2.19
S14	LE-ZnPc	2.27	2.25	2.25	2.25
S15	LE-ZnPc	2.27	2.26	2.25	2.25
T1	LE-ZnPc	1.21	1.21	1.21	1.20
T2	LE-ZnPc	1.22	1.23	1.23	1.22
T3	CT	1.47	1.38	1.35	1.34
T4	CT	1.50	1.40	1.37	1.34
T5	CT	1.53	1.41	1.39	1.38
T6	LE-C_{60}	1.82	1.79	1.78	1.77
T7	LE-C_{60}	1.83	1.79	1.78	1.78
T8	LE-C_{60}	1.83	1.80	1.79	1.78
T9	LE-C_{60}	2.05	1.99	1.98	1.97
T10	LE-C_{60}	2.05	1.99	1.98	1.97

Table S5. Calculated singlet and triplet energies of the $F_4ZnPc:C_{60}$ complex as a function of dielectric constant. The charge transfer (CT) and local excitation (LE) singlet F_4ZnPc states are shown in red and blue, respectively.

State	[eV]	ε=2	ε=3	ε=3.5	ε=4
S1	CT	1.50	1.39	1.36	1.34
S2	CT	1.50	1.39	1.36	1.34
S3	CT	1.57	1.45	1.41	1.39
S4	LE-C_{60}	2.21	2.16	2.14	2.12
S5	LE-C_{60}	2.22	2.16	2.14	2.13
S6	LE-C_{60}	2.22	2.17	2.15	2.14
S7	LE-C_{60}	2.23	2.17	2.15	2.14
S8	LE-C_{60}	2.23	2.18	2.16	2.15
S9	LE-C_{60}	2.23	2.19	2.17	2.16
S10	LE-C_{60}	2.25	2.19	2.17	2.16
S11	LE-C_{60}	2.25	2.20	2.18	2.17
S12	LE-C_{60}	2.25	2.21	2.19	2.18
S13	LE-C_{60}	2.26	2.21	2.19	2.18
S14	LE-F_4ZnPc	2.27	2.21	2.20	2.20
S15	LE-F_4ZnPc	2.27	2.22	2.21	2.21
T1	LE-F_4ZnPc	1.20	1.20	1.20	1.20
T2	LE-F_4ZnPc	1.20	1.21	1.21	1.21
T3	CT	1.50	1.41	1.38	1.36
T4	CT	1.54	1.44	1.41	1.38
T5	CT	1.57	1.44	1.42	1.40
T6	LE-C_{60}	1.83	1.79	1.78	1.77
T7	LE-C_{60}	1.83	1.79	1.78	1.78
T8	LE-C_{60}	1.83	1.80	1.79	1.78
T9	LE-C_{60}	2.05	2.00	1.98	1.97
T10	LE-C_{60}	2.05	2.00	1.98	1.97

Table S6. Calculated singlet and triplet energies of the $F_8ZnPc:C_{60}$ complex as a function of dielectric constant. The charge transfer (CT) and local excitation (LE) singlet F_8ZnPc states are shown in red and blue, respectively.

State	[eV]	ε=2	ε=3	ε=3.5	ε=4
S1	CT	1.61	1.52	1.49	1.47
S2	CT	1.62	1.52	1.49	1.47
S3	CT	1.70	1.58	1.55	1.52
S4	LE-C_{60}	2.21	2.15	2.13	2.12
S5	LE-C_{60}	2.22	2.16	2.14	2.13
S6	LE-C_{60}	2.22	2.16	2.14	2.13
S7	LE-C_{60}	2.22	2.16	2.15	2.13
S8	LE-C_{60}	2.24	2.18	2.17	2.15
S9	LE-C_{60}	2.24	2.19	2.17	2.16
S10	LE-C_{60}	2.25	2.19	2.17	2.16
S11	LE-C_{60}	2.25	2.19	2.18	2.17
S12	LE-C_{60}	2.26	2.21	2.19	2.18
S13	LE-C_{60}	2.26	2.21	2.19	2.18
S14	LE-F_8ZnPc	2.27	2.27	2.27	2.27
S15	LE-F_8ZnPc	2.29	2.28	2.27	2.27
T1	LE-F_8ZnPc	1.24	1.25	1.25	1.25
T2	LE-F_8ZnPc	1.25	1.26	1.26	1.27
T3	CT	1.62	1.52	1.50	1.48
T4	CT	1.65	1.56	1.53	1.51
T5	CT	1.69	1.58	1.54	1.52
T6	LE-C_{60}	1.82	1.79	1.78	1.77
T7	LE-C_{60}	1.83	1.79	1.78	1.78
T8	LE-C_{60}	1.83	1.79	1.78	1.78
T9	LE-C_{60}	2.04	1.99	1.98	1.97
T10	LE-C_{60}	2.04	1.99	1.98	1.97

Table S7. Calculated singlet and triplet energies of the $F_{16}ZnPc:C_{60}$ complex as a function of dielectric constant. The charge transfer (CT) and local excitation (LE) singlet $F_{16}ZnPc$ states are shown in red and blue, respectively.

State	[eV]	ε=2	ε=3	ε=3.5	ε=4
S1	CT	1.60	1.50	1.47	1.45
S2	CT	1.60	1.51	1.48	1.46
S3	CT	1.70	1.58	1.55	1.52
S4	LE-$F_{16}ZnPc$	2.16	2.14	2.13	2.12
S5	LE-$F_{16}ZnPc$	2.18	2.15	2.13	2.12
S6	LE-C_{60}	2.20	2.15	2.14	2.13
S7	LE-C_{60}	2.20	2.16	2.14	2.13
S8	LE-C_{60}	2.21	2.16	2.15	2.14
S9	LE-C_{60}	2.21	2.17	2.17	2.16
S10	LE-C_{60}	2.24	2.18	2.17	2.16
S11	LE-C_{60}	2.24	2.19	2.17	2.16
S12	LE-C_{60}	2.24	2.19	2.18	2.16
S13	LE-C_{60}	2.25	2.19	2.18	2.17
S14	LE-C_{60}	2.25	2.20	2.19	2.17
S15	LE-C_{60}	2.25	2.20	2.19	2.17
T1	LE-$F_{16}ZnPc$	1.18	1.18	1.19	1.19
T2	LE-$F_{16}ZnPc$	1.18	1.19	1.19	1.19
T3	CT	1.60	1.51	1.48	1.46
T4	CT	1.64	1.54	1.52	1.49
T5	CT	1.69	1.57	1.54	1.51
T6	LE-C_{60}	1.82	1.79	1.78	1.77
T7	LE-C_{60}	1.82	1.79	1.78	1.77
T8	LE-C_{60}	1.82	1.79	1.78	1.78
T9	LE-C_{60}	2.03	1.98	1.97	1.96
T10	LE-C_{60}	2.03	1.99	1.97	1.96

Table S8. Calculated molecular orbitals as a function of dielectric constant (ε). The lowest unoccupied molecular orbital (LUMO) and highest occupied molecular orbital (HOMO) levels were determined for isolated F_xZnPc and C_{60} as described above.

	ε	ZnPc	F_4ZnPc	F_8ZnPc	$F_{16}ZnPc$	C_{60}
LUMO	2	-2.43	-2.63	-2.92	-3.25	-2.95
	3	-2.65	-2.85	-3.13	-3.45	-3.19
	3.5	-2.71	-2.91	-3.19	-3.51	-3.25
	4	-2.76	-2.95	-3.23	-3.55	-3.30
HOMO	2	-5.41	-5.54	-5.90	-6.06	-6.61
	3	-5.20	-5.34	-5.69	-5.86	-6.36
	3.5	-5.14	-5.28	-5.64	-5.80	-6.29
	4	-5.10	-5.23	-5.59	-5.76	-6.23

Table S9. Calculated transition dipole moments and electronic coupling. The transition dipole moments between the local excitation (LE) singlet and ground state (GS) ($\mu_{LE\text{-}GS}$), difference between the dipole moments of the GS and charge transfer (CT) state ($\Delta\mu_{CT\text{-}GS}$), and electronic couplings between the GS and CT state ($V_{CT\text{-}GS}$), and between the CT and LE states ($V_{CT\text{-}LE}$) for the F_xZnPc:C_{60} complexes as calculated via the 2-state generalized Mulliken-Hush approach based on density functional theory (DFT) results at the LC-ωhPBE/6-31G** level of theory.

	ZnPc:C_{60}	F_4ZnPc:C_{60}	F_8ZnPc:C_{60}	$F_{16}ZnPc$:C_{60}
$\mu_{LE\text{-}GS}$ (D)	6.48	6.53	6.58	7.47
$\Delta\mu_{CT\text{-}GS}$ (D)	22.9	22.1	21.8	20.3
$V_{CT\text{-}GS}$ (eV)	65	75	84	101
$V_{CT\text{-}LE}$ (eV)	165	102	144	116

Table S10. Density functional theory (DFT) derived zero-field splitting parameters.

	B3LYP/6-31G**		PBE0/6-31G**		B3LYP/def2-TZVP		B3LYP/EPR-II def2-TZVP for Zn	
	D [cm^{-1}]	E/D	D [cm^{-1}]	E/D	D [cm^{-1}]	E/D	D [cm^{-1}]	E/D
ZnPc	2.676	0.003	2.674	0.005	0.961	0.016	0.379	0.022
F_4ZnPc	2.618	0.003	2.582	0.003	0.985	0.014	0.380	0.020
F_8ZnPc	2.618	0.002	2.580	0.003	0.797	0.024	0.385	0.027
F_{16}ZnPc	2.597	0.002	2.553	0.003	0.765	0.024	0.406	0.006

Table S11. Relaxation energies (λ_{rel}) of the F_xZnPc and C_{60} molecules in the charged state and intramolecular reorganization energies for the charge transfer (CT) state of the F_xZnPC:C_{60} complex ($\lambda_{intra\text{-}CT}$). The relaxation energies were obtained from the adiabatic potential surfaces of the neutral and charged states and from a normal-mode analysis. The charged states of the F_xZnPc and C_{60} components correspond to the cation and anion states, respectively.

[meV]	Adiabatic potential surface	Normal mode
λ_{rel} (ZnPc)	21	22
λ_{rel} (F_4ZnPc)	30	31
λ_{rel} (F_8ZnPc)	37	39
λ_{rel} (F_{16}ZnPc)	51	53
λ_{rel} (C_{60})	76	75
$\lambda_{intra\text{-}CT}$ (ZnPc:C_{60})	97	97
$\lambda_{intra\text{-}CT}$ (F_4ZnPc:C_{60})	106	106
$\lambda_{intra\text{-}CT}$ (F_8ZnPc:C_{60})	113	114
$\lambda_{intra\text{-}CT}$ (F_{16}ZnPc:C_{60})	127	128

Table S12. Parameters used in three-state model fitting of the dilute ZnPc:C_{60} and F_4ZnPc:C_{60} blends. Charge transfer (CT) and local excitation (LE) energies (E_{CT} and E_{LE}), energy differences between LE and CT states (ΔE_{CT}), transition dipole moments to the LE states ($\mu_{LE\text{-}GS}$), state dipole moments of the CT states (μ_{CT}), electronic couplings between CT and ground states (GS; $V_{CT\text{-}GS}$) and between CT and LE states ($V_{LE\text{-}CT}$), frequencies of low- and high-energy vibrational modes (LF and HF), and vibronic coupling constants (g) corresponding to the LE or CT state for the LF or HF vibrational mode.

	ZnPc:C_{60}	F_4ZnPc:C_{60}
E_{CT} (eV)	1.38	1.45
E_{LE} (eV)	1.69	1.65
ΔE_{CT} (eV)	0.31	0.20
$^{\#}\mu_{LE\text{-}GS}$ (D)	6.48	6.53
$^{\#}\mu_{CT}$ (D)	22.9	22.1
$^{\#}V_{CT\text{-}GS}$ (eV)	0.065	0.075
$V_{LE\text{-}CT}$ (eV)	0.090	0.081
$^{\#}$LF (cm^{-1})	100	100
$^{\#}$HF (cm^{-1})	1200	1200
g_{LF}^{LE}	2.74	2.74
g_{HF}^{LE}	0.56	0.56
g_{LF}^{CT}	4.18	5.00
g_{HF}^{CT}	0.85	1.02

$^{\#}$ These are fixed during fitting process. The others are fitted parameters.

Table S13. Best-fit values obtained from fitting the time-resolved electron paramagnetic resonance (trEPR) spectra of neat F_xZnPc films. For each film, the populating mechanism of the triplet states, with the respective triplet sublevels populations [p_x p_y p_z], and the zero-filed splitting (ZFS) parameters ([D E]) are reported. Intersystem crossing (ISC) populations order (p_1 p_2 p_3) is from low-to-high energy zero-field states. Only gaussian broadening was considered for all triplets to avoid over-parameterizing the fits. The relative weight of ISC and back electron transfer (BET) triplets is reported in the last column. Finally, for the simulations of ISC triplets of ZnPc and F_4ZnPc, a preferential ordering contribution is needed, which suggests preferential ordering of the crystallites with respect to the substrate.

	Triplet Species	[D E] (MHz)	[p_x p_y p_z]	LW_{ISC} (mT)	[p_{-1} p_0 p_{+1}]	LW_{BET} (mT)	$weight_{ISC}$: $weight_{BET}$	Order
ZnPc	ISC	[387 36]	[0.19 0.33 0.48]	2.52			1:0	0.64
F_4ZnPc	ISC	[516 97]	[0.21 0.34 0.45]	1.9			1:0	0.09
F_8ZnPc	ISC+BET	[535 87]	[0.47 0.07 0.46]	3.4	[1 0 1]	3.2	0.22:0.78	
$F_{16}ZnPc$	ISC	[505 150]	[0.39 0.36 0.25]	3.5			1:0	

SUPPLEMENTARY NOTE 1: Grazing Incidence Wide-Angle X-Ray Scattering Results

Grazing incidence wide-angle X-ray (GIWAXS) scattering was carried out to characterize the microstructure and crystallinity of the films. Figure S1 shows the 2D reciprocal space maps of the investigated systems. All neat films show crystalline features, in particular a strong out-of-plane peak indexed as the (200) peak, in line with previous work.[1] This indicates that the preferential orientation of the F_xZnPc molecules is with their long axes aligned roughly parallel to the out-of-plane axis direction. The peak broadening along the azimuthal angle indicates a significant spread in the orientation of crystalline domains and is seen for all neat films. At least one in-plane scattering feature attributed to the $\pi - \pi$ stacking (010) peak is also seen, along with a secondary in-plane scattering feature for neat ZnPc. The positions of the peaks are plotted in Figure S2.

Increased fluorination is concomitant with increased spacing along the long axis stacking direction. F_8ZnPc and $F_{16}ZnPc$ show a decreased $\pi - \pi$ stacking d-spacing compared to ZnPc and F_4ZnPc.

The low-donor films show no crystalline features attributable to C_{60} and only faint (200) scattering features around the out-of-plane direction. The lack of scattering from C_{60}, which is the dominant molecular species in the thin films, indicates that the presence of the F_xZnPc interrupts C_{60} packing in all cases. Thus, as stated in the main text, at low concentrations all donor molecules are surrounded by acceptors and donor aggregation is largely suppressed.

SUPPLEMENTARY NOTE 2: Contributions to the Photocurrent

The short-circuit current density (J_{SC}) is governed by the active-layer absorbance, exciton separation efficiency, and charge collection efficiency. Upon increasing donor fluorination, active-layer absorbance, as observed via the extinction coefficient of the blends, remains largely unchanged (Figure S5a). With nominally identical device stacks and active-layer thicknesses of 50 nm, changes in the charge collection efficiency, potentially reflected in a small relative decrease in fill factor between the dilute ZnPc and F_{16}ZnPc blends (Table S1), are not enough to explain the ~ 80% drop in J_{SC} with increasing fluorination. Instead, the J_{SC} is primarily limited by poor exciton dissociation caused by the reduced energetic offset at the donor-acceptor interface.

SUPPLEMENTARY NOTE 3: Determination of Energy Losses

Energy losses are calculated by comparing a reference energy (E_{ref}) to the open circuit voltage ($q\Delta V = E_{ref} - qV_{oc}$). In the energetic state picture,[10] E_{ref} is defined as the energy of the local excited state (E_{LE}). From this, the driving force for CT is determined as the difference between E_{LE} and the CT state energy ($\Delta E_{CT} = E_{LE} - E_{CT}$). We determined E_{LE} and E_{CT} via gaussian fitting. The radiative saturation current density (J_0^{rad}) is determined via integration of the EQE, according to $J_0^{rad} = q\int_0^{\infty} EQE \cdot \phi_{BB} dE$, where ϕ_{BB} is the black body spectrum. By comparing J_0^{rad} to the short-circuit current density (J_{sc}), the radiative energy limit of the open circuit voltage (qV_{oc}^{rad}) is determined as $qV_{oc}^{rad} = k_B T \ln\left(\frac{J_{sc}}{J_0^{rad}}\right)$, where k_B is the Boltzmann constant and T is the temperature. qV_{oc}^{rad} does not require the EQE to follow a step function but assumes that only radiative recombination occurs. Importantly, to determine qV_{oc}^{rad}correctly, the EQE needs to be measured with sufficient dynamic range. To ensure that qV_{oc}^{rad} saturates, the EQE

is often extended beyond the limit of the measurement setup,[11] in our case using the gaussian functions derived from EQE fitting (Figure S9). All presented energy losses were calculated for a minimum energy of 0.5 eV, which safely falls within the saturation limit. From this, radiative energy losses are calculated as $\Delta qV_{oc}^{rad} = E_{CT} - qV_{oc}^{rad}$. Finally, non-radiative energy losses (qV_{oc}^{nr}) are determined via $qV_{oc}^{nr} = qV_{oc}^{rad} - qV_{oc} = -k_b T$ ln (EQE_{EL}). Here, $EQE_{EL}$is the electroluminescence quantum efficiency, which depends on e.g., the fraction of radiative recombination events (χ) and the photoluminescence quantum efficiency (ϕ_{PL}).[12]

Looking at the trends of nonradiative energy losses vs. E_{CT} of the devices (Figure S12a), we observe decreasing non-radiative losses with increasing E_{CT}, as predicted by the energy gap law.[13] Comparing this to the results presented in the main paper, namely the increase in triplet formation observed for increasing donor fluorination, we note that increasing triplet formation decreases the fraction of radiative recombination, while the energy gap law increases the photoluminescence quantum yield. EQE_{EL}, and with this non-radiative energy losses, are derived from the balance between these two contributions. We model this interplay between the different factors influencing qV_{oc}^{nr} via equations 1 and 2 in Gillett et al. (Figure S12c).[12] Here, we assume a photon out-coupling efficiency of 0.3, a charge balance factor of 1, and a temperature of 300 K.[12] As seen in Figure S12c, qV_{oc}^{nr} is highest when the fraction of radiative recombination events is low. With increasing χ, qV_{oc}^{nr} decreases, most significantly between $\chi = 0 - 0.2$ and more gradually for $\chi > 0.2$. Similarly, qV_{oc}^{nr} decreases with increasing photoluminescence quantum efficiency. In our case, we observe a 0.5 eV reduction in qV_{oc}^{nr} when ϕ_{PL} increases from 0.4% to 2%.

In summary, while χ is influenced by the presence of triplet recombination pathways (as studied in the main manuscript), ϕ_{PL} also depends on the vibration-induced direct recombination from the CT to the ground state. The total energy losses of a specific material

system arise from the combination of all these factors and are often challenging to disentangle. Triplet recombination has recently gained significant interest in literature[12,14–18] as an important (and avoidable) contribution to the total energy losses. Gillett at al. estimated the potential for reducing energy losses by eliminating triplet recombination pathways to be 0.6 eV [12], which is about 10% of the total energy losses commonly measured for organic solar cells, specifically those with large energetic offsets between singlet and CT state energies. However, triplet-mediated losses are by no means the only (or often most dominant) contribution to energy losses. In fact, for the material systems studied by Gillett et al. (primarily based on NFAs), non-radiative losses were still found to reach $0.2 - 0.3$ eV, even when both geminate and non-geminate triplet recombination pathways were suppressed.

SUPPLEMENTARY NOTE 4: Discussion of the DFT Results

We determined the geometries of isolated F_xZnPc:C_{60} complexes (Figure S13) and used time dependent DFT (TDDFT) to calculate their excited state properties. The natural transition orbital (NTO) analysis (Figure S14) indicates that the lowest three excited singlet states in all complexes are CT states between the donor and acceptor. These three CT states are quasi-degenerate due to the three-fold orbital degeneracy of the C_{60} LUMO. The CT states are followed by ten quasi-degenerate C_{60} singlet states and two degenerate F_xZnPc singlet states, which are all in near resonance (Tables S4-S7). For the sake of comparison, we note that measurements performed on C_{60} and ZnPc molecules embedded in matrixes of light noble gas atoms indicate that the 0-0 transition of the lowest excited singlet state has an energy of 1.94 eV and 1.91 eV for C_{60} [19] and ZnPc,[20] respectively. While the TDDFT estimates of these energies are larger than the experimental values, the TDDFT calculations correctly predict that the lowest excited states of isolated C_{60} and ZnPc molecules have comparable energies.

Comparing the calculated CT state energies, we observe that the four complexes can be grouped into two pairs, ZnPc:C_{60} with F_4ZnPc:C_{60}, and F_8ZnPc:C_{60} with F_{16}ZnPc:C_{60}, since the E_{CT} energy difference within each pair is smaller than between pairs (Tables S4-S7). This pairing matches the same pairing observed in terms of device performance. More specifically, when considering a dielectric medium with ε=4 (approximately the static dielectric constant of a neat C_{60} film), the TDDFT-derived CT state energies span a range of 1.3 eV (ZnPc:C_{60}) to 1.45 eV (F_{16}ZnPc:C_{60}).

To provide an alternative estimate of E_{CT}, we use a simplified but widely employed method [21–24] and represent the CT state energy as the sum of E_{CS} and the hole-electron electrostatic interaction energy (E_{el}):

$$E_{CT} = E_{CS} + E_{el} \qquad (S1)$$

Our DFT calculations of the donor-acceptor complexes (containing one F_xZnPc molecule and one C_{60} molecule) show that the center-to-center distance between donor and acceptor molecules is similar for all four material systems. Given the low donor content, we assume that the dielectric constant of the blends resembles that of neat C_{60}. Based on these considerations, we estimate a value of -0.5 eV for the E_{el} of all studied F_xZnPc:C_{60} blends. This value agrees with the E_{el} values calculated previously[24] for a range of donor-acceptor systems, including ZnPc:C_{60}. Estimating E_{CS} via the energies of the frontier molecular orbitals of the donors and acceptor, we then obtain:

$$E_{CT} = [E_{\mathrm{LUMO}}(C_{60}) - E_{\mathrm{HOMO}}(F_X ZnPc)] + E_{el} \qquad (S2)$$

Using the E_{LUMO} and E_{HOMO} values calculated for F_xZnPc and C_{60} molecules embedded into a dielectric medium with ε=4 (see Table S8), we estimate the following CT energies for the investigated systems: ZnPc:C_{60} (1.30 eV) with F_4ZnPc:C_{60} (1.43 eV), and F_8ZnPc:C_{60} (1.79 eV) with F_{16}ZnPc:C_{60} (1.96 eV). While this "electrostatic" approach following equations *S1* and *S2* yields E_{CT} energies of ZnPc:C_{60} and F_4ZnPc:C_{60} that closely match the values

determined via TDDFT and EQE-fitting, the derived E_{CT} of F_8ZnPc:C_{60} and F_{16}ZnPc:C_{60} are found to be larger than predicted by other two approaches.

In summary, while the energy difference between the CS and CT state (ΔE_{CS}) is found to increase when determining E_{CT} via TDDFT or EQE-fitting (see Table 1 in the main text), the "electrostatic" method assumes that ΔE_{CS} is constant. This discrepancy can be attributed to the simplicity of the electrostatic model that, in particular, neglects that electronic polarization energies (that define E_{LUMO} and E_{HOMO}) are also dependent on the distance between electrons and holes.

Finally, our DFT calculations indicate that the energies of the lowest triplet state (T_1) in the F_xZnPc series are about 1.2 eV. This value compares well with the experimental value of 1.13 eV measured for the triplet states of ZnPc in solution.[16,25,26] The DFT estimate for T_1 in C_{60} is about 1.77 eV, which is slightly larger than the measured value of 1.6 eV for C_{60} in a noble gas matrix[27] or 1.50 eV measured in C_{60} thin films.[28] In any case, the DFT calculations and the experimental data both confirm that the T_1 of F_xZnPc is the lowest excited state in the blends, being lower in energy than the T_1 of C_{60}.

SUPPLEMENTARY NOTE 5: Three-State Vibronic Model Fitting

The low-energy external quantum efficiency (EQE) spectra of the F_xZnPc:C_{60} complexes were simulated by means of a three-state dynamic vibronic model we recently reported.[29] All parameters used in the three-state model study are collected in Table S12.

SUPPLEMENTARY NOTE 6: Electron Paramagnetic Resonance Analysis of neat F_xZnPc

We carried out time-resolved electron paramagnetic resonance (trEPR) at 80 K by using 532 nm laser excitation on the neat and dilute donor films (Figures S17 – S21). The trEPR measurements of the dilute donor films are discussed in detail in the main paper. Investigating the trEPR of the neat films, all spectra show two main species: (1) a spectrally narrow signal in neat absorption or emission at ≈345 mT, which can be attributed to photogenerated charges, most likely radical pairs, and (2) a broad signal ranging from about 320 to 370 mT which can be attributed to local F_xZnPc triplet states (Figure S19). The weak signal of the photogenerated radical pairs (magnified in Figure S18) highlights that charges are generated following laser excitation. Even without an acceptor molecule, the neat films show weak but appreciable photoactivity (Figure S4).

Notably, the typical emission (*e*) and absorption (*a*) patterns of *eaea* or *aeae* of spin-correlated radical pairs are not observable. This can be due to several reasons, e.g. (1) rapid spin-relaxation,[30] (2) distortions due to a sequential electron transfer process,[31] (3) alternative spin polarization mechanisms,[32] or more trivially (4) a weak signal-to-noise ratio. An analysis of the spin-polarization of the radical pairs is beyond the scope of this manuscript and we therefore focus the following discussion only on the triplet state signal. In this regard, the full 2D trEPR contour plots are reported in Figure S17, the 1D trEPR spectra at three relevant delays from the initial laser excitation are shown in Figure S18, and the best-fit simulations of the triplet spectra (1 µs after laser excitation) including their calculated values are reported in Figure S19 and Table S13, respectively and discussed in the following.

A comparison of our results for the neat ZnPc film with those reported by Barbon et al. for ZnPc powder highlights a lower D value in our film.[33] Since, in first approximation, the D value is inversely proportional to the cubed delocalization r of the triplet state ($D \sim r^{-3}$), [34] a lower D value suggests that triplet excitons in our film are slightly more delocalized. In this

regard, it is worth noting that the dielectric environment can play a pivotal role on the triplet wavefunction and in turn on the zero-field splitting values.[35,36]Therefore, partial exciton delocalization to nearby molecules in the evaporated ZnPc film could explain the lower D value.[37] Regarding the F_xZnPc films, their D values are larger (505-535 MHz) than for non-fluorinated ZnPc (387 MHz). This trend is in good agreement with calculations, which show higher ZFS values for the fluorinated ZnPc molecules (Table S10).

Notably, the spin populations of the triplet sublevels show that in all neat donor films, the triplet states are typical for intersystem crossing (ISC) triplets, as opposed to triplets populated via back electron transfer (BET). This result can be rationalized considering that in the neat films most of the singlet excitons do not dissociate and undergo ISC before recombining to the ground state.[12,38] The F_8ZnPc film shows an additional contribution of a geminate BET triplet in addition to ISC, which suggests that after weak charge photogeneration the charges may geminately recombine back to a low-lying triplet state in this film. This process is energetically favorable since the triplet state is lower in energy compared to the CT state (see Table S6). Furthermore, while the spectrum of the neat F_4ZnPc film does not show any contribution of BET triplets at t = 1 μs, the significant spectral change of the trEPR signal at later times suggests that BET to a low-lying triplet most likely occurs at later times in neat F_4ZnPc. Notably, similar BET triplet mechanisms are not unusual for neat films of pure D or A molecules, as previously reported for e.g. PC_{60}BM films.[39]

SUPPLEMENTARY NOTE 7: Spin-Polarization of BET Triplets

As discussed in detail in the literature [32,40,41], geminate BET contributions can be understood in the framework of the “spin correlated radical pair (SCRP) mechanism”. Standard geminate recombination pathways start from the CT state, which in EPR spectroscopy is termed the SCRP due to the significant magnetic exchange and spin-spin dipolar interactions between the

two unpaired spins. In fact, the radical pair is composed of a hole mainly localized on the donor and an electron mainly localized on the acceptor, that interact through the exchange and spin-spin dipolar interactions and generate four spin states, which for clarity we call $^1CT_0$, $^3CT_0$, $^3CT_{+1}$ and $^3CT_{-1}$. The $^1CT_0$ and $^3CT_0$ (m_s=0) spin sublevels of the SCRP are "mixed" together due to hyperfine and electron Zeeman interactions.

In this context, two distinct pathways can lead to the presence of BET triplet polarization patterns: (1) The SCRP $^3CT_0$ sublevel formed by mixing with the $^1CT_0$ undergoes a spin-allowed BET to an energetically low-lying molecular triplet T_0 state, generating an excess spin population in the T_0. This results in a spin polarization pattern of *eaaeea* (D<0) or *aeeaae* (D>0) for the triplet exciton.[40] (2) The mixing of the $^1CT_0$ and $^3CT_0$ SCRP sublevels opens a spin-allowed recombination pathway for $^3CT_0$ to the S_0 ground state via $^1CT_0$. This process results in an excess population remaining in the $^3CT_{+1}$ and $^3CT_{-1}$, which undergo a spin-allowed BET to the molecular triplet T_{+1} and T_{-1} sublevels, generating an excess spin population in the T_{+1} and T_{-1}. This results in a spin polarization pattern of *eaaeea* (D>0) or *aeeaae* (D<0) for the triplet exciton.[32]

Interestingly, assuming a positive sign of D for the donor T_1 as done for similar systems in the literature, and suggested by our DFT calculations, we can conclude that in our case the main polarization mechanism is the second one.[42]

SUPPLEMENTARY NOTE 8: Partial Preferential Order of Triplet States

Due to the anisotropic spin-spin dipolar interaction between two electron spins, triplet states are sensitive to how their orientation compares to the external magnetic field.[38] As a result, they can be used to deduce information about the orientation of molecules with respect to a given reference system. In a sample with some degree of molecular order, the trEPR spectrum

deviates from the "random" powder spectrum and a non-uniform distribution of molecular orientations, $P(\theta, \phi)$, should be considered to reproduce the spectral shape.[43]

In our manuscript, given the lack of donor aggregation in the dilute blends (discussed in the microstructure section above), we do not observe any contributions of partial order in the simulations presented in Figure 3 in the main paper. However, for the neat ZnPc and F_4ZnPc films (Figure S19), we introduce an order parameter to correctly reproduce the outer shoulders of the triplet spectrum (corresponding to the D_z position) which could not be fully accounted for using full powder averaging. In order to do this, we used the Easyspin command *Exp.Ordering* which specifies the nature (positive values signify a molecular director parallel to B_0, negative values signify a molecular director perpendicular to B_0) and the degree of ordering.[44]

We note that in our simulations, we introduce the molecular order as a phenomenological parameter to reproduce the spectra of these two neat films without studying the angular dependence of the trEPR spectra in detail. Nevertheless, the need to introduce partial orientation for these two spectra may suggest that the neat ZnPc and F_4ZnPc films show a higher degree of molecular order compared to the F_8ZnPc and F_{16}ZnPc films.

SUPPLEMENTARY REFERENCES

1. Derrien, T. L. *et al.* Interfacial rearrangements and strain evolution in the thin film growth of ZnPc on glass. *Phys Rev Mater* 6, 033401 (2022).
2. Brendel, M. *et al.* The effect of gradual fluorination on the properties of FnZnPc thin films and FnZnPc/C60 bilayer photovoltaic cells. *Adv Funct Mater* 25, 1565–1573 (2015).
3. Brinkmann, H. *et al.* Fluorinated phthalocyanines as molecular semiconductor thin films. *Physica Status Solidi (A) Applications and Materials Science* 205, 409–420 (2008).
4. Burkhard, G. F., Hoke, E. T. & McGehee, M. D. Accounting for interference, scattering, and electrode absorption to make accurate internal quantum efficiency measurements in organic and other thin solar cells. *Advanced Materials* 22, 3293–3297 (2010).
5. Vandewal, K., Tvingstedt, K., Gadisa, A., Inganäs, O. & Manca, J. V. On the origin of the open-circuit voltage of polymer-fullerene solar cells. *Nat Mater* 8, 904–909 (2009).
6. Vandewal, K., Tvingstedt, K., Gadisa, A., Inganäs, O. & Manca, J. V. Relating the open-circuit voltage to interface molecular properties of donor:acceptor bulk heterojunction solar cells. *Phys Rev B Condens Matter Mater Phys* (2010) doi:10.1103/PhysRevB.81.125204.
7. Jungbluth, A., Kaienburg, P. & Riede, M. Charge transfer state characterization and voltage losses of organic solar cells. *Journal of Physics: Materials* 5, 024002 (2022).
8. Li, T. Y. *et al.* Boron dipyrromethene (BODIPY) with: Meso -perfluorinated alkyl substituents as near infrared donors in organic solar cells. *J Mater Chem A Mater* 6, 18583–18591 (2018).
9. Nikolis, V. C. *et al.* Field Effect versus Driving Force: Charge Generation in Small-Molecule Organic Solar Cells. *Adv Energy Mater* 10, (2020).
10. Vandewal, K., Benduhn, J. & Nikolis, V. C. How to determine optical gaps and voltage losses in organic photovoltaic materials. *Sustainable Energy and Fuels* vol. 2 538–544 Preprint at https://doi.org/10.1039/c7se00601b (2018).
11. Kaienburg, P. *et al.* Assessing the Photovoltaic Quality of Vacuum-Thermal Evaporated Organic Semiconductor Blends. *Advanced Materials* 34, (2022).
12. Gillett, A. J. *et al.* The role of charge recombination to triplet excitons in organic solar cells. *Nature* 597, 666–671 (2021).
13. Benduhn, J. *et al.* Intrinsic non-radiative voltage losses in fullerene-based organic solar cells. *Nat Energy* 2, (2017).
14. Privitera, A. *et al. Combining Optical and Magnetic Resonance Spectroscopies to Probe Charge Recombination via Triplet Excitons in Organic Solar Cells*.
15. Chen, Z. *et al.* Triplet exciton formation for non-radiative voltage loss in high-efficiency nonfullerene organic solar cells. *Joule* 5, 1832–1844 (2021).
16. Benduhn, J. *et al.* Impact of Triplet Excited States on the Open-Circuit Voltage of Organic Solar Cells. *Adv Energy Mater* 8, (2018).
17. Privitera, A. *et al.* Geminate and Nongeminate Pathways for Triplet Exciton Formation in Organic Solar Cells. (2022) doi:10.1002/aenm.202103944.
18. Köhler, A. & Bässler, H. What controls triplet exciton transfer in organic semiconductors? *J. Mater. Chem.* 21, (2011).
19. Orlandi, G. & Negri, F. Electronic states and transitions in C60 and C70 fullerenes. *Photochemical and Photobiological Sciences* 1, 289–308 (2002).

20. Vancott, T. C. *et al. Magnetic Circular Dichroism and Absorption Spectrum of Zinc Phthalocyanine in an Argon Matrix between 14 700 and 74 000 Cm"1. J. Phys. Chem* vol. 93 https://pubs.acs.org/sharingguidelines (1989).
21. Zheng, Z., Egger, D. A., Brédas, J.-L., Kronik, L. & Coropceanu, V. Effect of Solid-State Polarization on Charge-Transfer Excitations and Transport Levels at Organic Interfaces from a Screened Range-Separated Hybrid Functional. *J Phys Chem Lett* 8, 3277–3283 (2017).
22. Zheng, Z., Tummala, N. R., Wang, T., Coropceanu, V. & Brédas, J. L. Charge-Transfer States at Organic–Organic Interfaces: Impact of Static and Dynamic Disorders. *Adv Energy Mater* 9, (2019).
23. Yi, Y., Coropceanu, V. & Brédas, J.-L. Exciton-Dissociation and Charge-Recombination Processes in Pentacene/C60 Solar Cells: Theoretical Insight into the Impact of Interface Geometry. *J Am Chem Soc* 131, 15777–15783 (2009).
24. Scholz, R. *et al.* Quantifying charge transfer energies at donor–acceptor interfaces in small-molecule solar cells with constrained DFTB and spectroscopic methods. *Journal of Physics: Condensed Matter* 25, 473201 (2013).
25. Savolainen, J., van der Linden, D., Dijkhuizen, N. & Herek, J. L. Characterizing the functional dynamics of zinc phthalocyanine from femtoseconds to nanoseconds. *J Photochem Photobiol A Chem* 196, 99–105 (2008).
26. Vincett, P. S., Voigt, E. M. & Rieckhoff, K. E. Phosphorescence and fluorescence of phthalocyanines. *J Chem Phys* 55, 4127–4131 (1971).
27. Orlandi, G. & Negri, F. Electronic states and transitions in C60 and C70 fullerenes. *Photochemical and Photobiological Sciences* 1, 289–308 (2002).
28. Kazaoui, S. *et al.* Comprehensive analysis of intermolecular charge-transfer excited states in C60 and C70 films.
29. Chen, X. K., Coropceanu, V. & Brédas, J. L. Assessing the nature of the charge-transfer electronic states in organic solar cells. *Nat Commun* 9, (2018).
30. Miura, T., Aikawa, M. & Kobori, Y. Time-Resolved EPR Study of Electron–Hole Dissociations Influenced by Alkyl Side Chains at the Photovoltaic Polyalkylthiophene:PCBM Interface. *J Phys Chem Lett* 5, 30–35 (2014).
31. Niklas, J. *et al.* Photoinduced Dynamics of Charge Separation: From Photosynthesis to Polymer-Fullerene Bulk Heterojunctions. *Journal of Physical Chemistry B* 119, 7407–7416 (2015).
32. Franco, L. *et al.* Time-resolved EPR of photoinduced excited states in a semiconducting polymer/PCBM blend. *Journal of Physical Chemistry C* 117, 1554–1560 (2013).
33. Barbon, A., Brustolon, M. & van Faassen, E. E. Photoexcited spin triplet states in zinc phthalocyanine studied by transient EPR. *Physical Chemistry Chemical Physics* 3, 5342–5347 (2001).
34. Richert, S., Tait, C. E. & Timmel, C. R. Delocalisation of photoexcited triplet states probed by transient EPR and hyperfine spectroscopy. *Journal of Magnetic Resonance* 280, 103–116 (2017).
35. Minaev, B., Loboda, O., Vahtras, O., Ruud, K. & Ågren, H. Solvent effects on optically detected magnetic resonance in triplet spin labels. *Theor Chem Acc* 111, 168–175 (2004).
36. Gillett, A. J. *et al.* Dielectric control of reverse intersystem crossing in thermally activated delayed fluorescence emitters. *Nat Mater* 21, 1150–1157 (2022).
37. Rawat, N. *et al.* Macroscopic Molecular Ordering and Exciton Delocalization in Crystalline Phthalocyanine Thin Films. *J Phys Chem Lett* 6, 1834–1840 (2015).

38. Biskup, T. Structure-function relationship of organic semiconductors: Detailed insights from time-resolved EPR spectroscopy. *Front Chem* 7, (2019).
39. Righetto, M. *et al.* Engineering interactions in QDs-PCBM blends: A surface chemistry approach. *Nanoscale* 10, 11913–11922 (2018).
40. Thomson, S. A. J. *et al.* Charge Separation and Triplet Exciton Formation Pathways in Small-Molecule Solar Cells as Studied by Time-Resolved EPR Spectroscopy. *Journal of Physical Chemistry C* 121, 22707–22719 (2017).
41. Buckley, C. D., Hunter, D. A., Hore ', P. J. & Mclauchlan, K. A. *Electron Spin Resonance of Spin-Correlated Radical Pairs*. vol. 135 (1987).
42. Ikoma, T., Akiyama, K., Tero-Kubota, S. & Ikegami, Y. *Time-Resolved ESR Studies on the Excited Triplet States and Photoenolization of 2-Methylacetophenone and Related Molecules*. *J. Phys. Chem* vol. 93 https://pubs.acs.org/sharingguidelines (1989).
43. Biskup, T. *et al.* Ordering of PCDTBT Revealed by Time-Resolved Electron Paramagnetic Resonance Spectroscopy of Its Triplet Excitons. *Angewandte Chemie International Edition* 54, 7707–7710 (2015).
44. Stoll, S. & Schweiger, A. EasySpin, a comprehensive software package for spectral simulation and analysis in EPR. *Journal of Magnetic Resonance* 178, 42–55 (2006).
