# Supplementary material for: Limiting factors for charge generation in low-offset fullerene-based organic solar cells
Source: Nat Commun. 2024 Jun 28;15:5488. doi: 10.1038/s41467-024-49432-5 (PMC11213929; doi:10.1038/s41467-024-49432-5)
Supplement: Supplementary file 3 — Solar Cells Reporting Summary [file 41467_2024_49432_MOESM3_ESM.pdf]

nature portfolio

Corresponding author(s): Prof. Moritz Riede

Solar Cells Reporting Summary

Nature Portfolio wishes to improve the reproducibility of the work that we publish. This form is intended for publication with all accepted papers reporting the characterization of photovoltaic devices and provides structure for consistency and transparency in reporting. Some list items might not apply to an individual manuscript, but all fields must be completed for clarity.

For further information on Nature Research policies, including our data availability policy, see Authors & Referees.

Experimental design

Please check the following details are reported in the manuscript, and provide a brief description or explanation where applicable.

1. Dimensions

Item	Yes/No	Description / Explanation
Area of the tested solar cells	☒ Yes ☐ No	The pixel area (0.08 cm^2) is included under "Sample Fabrication Details" in the Methods section in the main paper. / *Explain why this information is not reported/not relevant.*
Method used to determine the device area	☒ Yes ☐ No	The pixel area is determined by the geometric overlap between the patterned ITO substrate, the organic layers, and the metal electrodes. This information is included under "Sample Fabrication Details" in the Methods section in the main paper. / *Explain why this information is not reported/not relevant.*

2. Current-voltage characterization

Item	Yes/No	Description / Explanation
Current density-voltage (J-V) plots in both forward and backward direction	☒ Yes ☐ No	Although hysteresis is not present in our OSC devices, both forward and backward scans are plotted in Figure S3 in the Supplementary Information.
Voltage scan conditions	☒ Yes ☐ No	The J-V scan conditions are detailed in the Methods section of the main paper All measurements were performed without dwell time. / *Explain why this information is not reported/not relevant.*
Test environment	☒ Yes ☐ No	The solar cells were encapsulated in a nitrogen-environment as detailed in the Methods section. / *Explain why this information is not reported/not relevant.*
Protocol for preconditioning of the device before its characterization	☐ Yes ☒ No	*Provide a description of the protocol.* / No preconditioning was performed before sample characterization.
Stability of the J-V characteristic	☐ Yes ☒ No	*Provide a description of the method used. The stability of the J-V characteristic can be verified with time evolution of the maximum power point or with the photocurrent at maximum power point; see ref. 5 for details.* / Stability is beyond the scope of this manuscript.

3. Hysteresis or any other unusual behaviour

Item	Yes/No	Description / Explanation
Description of the unusual behaviour observed during the characterization	☐ Yes ☒ No	*Provide a description of hysteresis or any other unusual behaviour observed during the characterization.* / No unusual behavior was observed in the J-V characteristics. Furthermore, our solar cells do not display hysteresis.
Related experimental data	☐ Yes ☒ No	*Provide a description of the related experimental data.* / Not relevant to this work.

4. Efficiency

Item	Yes/No	Description / Explanation
External quantum efficiency (EQE) or incident photons to current efficiency (IPCE)	☒ Yes ☐ No	EQE spectra are shown both in the main paper and in the Supplementary Information. / *Explain why this information is not reported/not relevant.*

A comparison between the integrated response under the standard reference spectrum and the response measure under the simulator

☐ Yes ☒ No

State where this information can be found in the text.

No high efficiency devices are presented in this paper.

For tandem solar cells, the bias illumination and bias voltage used for each subcell

☐ Yes ☒ No

Provide a description of the measurement conditions.

No tandem devices are presented in this paper.

5. Calibration

Light source and reference cell or sensor used for the characterization

☒ Yes ☐ No

Experimental details (including information on light sources and reference diodes) are provided in the Methods section in the main paper.

Explain why this information is not reported/not relevant.

Confirmation that the reference cell was calibrated and certified

☒ Yes ☐ No

Information on calibration of the J-V setup is provided in the Methods section of the main paper.

Explain why this information is not reported/not relevant.

Calculation of spectral mismatch between the reference cell and the devices under test

☐ Yes ☒ No

Provide a value of the spectral mismatch and/or a description of how it has been taken into account in the measurements.

Since our manuscript focuses on relative rather than absolute comparisons, and all presented donor molecules show strong similarities in their absorption profiles, no spectra mismatch was performed. Spectral mismatch can be added upon request.

6. Mask/aperture

Size of the mask/aperture used during testing

☐ Yes ☒ No

Report the size of the mask/aperture.

Measurements were performed without masking due to the well defined pixel area.

Variation of the measured short-circuit current density with the mask/aperture area

☐ Yes ☒ No

Report the difference in the short-circuit current density values measured with the mask and aperture area.

Not applicable to this work.

7. Performance certification

Identity of the independent certification laboratory that confirmed the photovoltaic performance

☐ Yes ☒ No

Identify the independent certification laboratory.

No high efficiency claims were made in this manuscript. No certification of the low efficiency organic solar cells presented in this work is needed.

A copy of any certificate(s)

☐ Yes ☒ No

Certificate copies should be provided in the Supplementary information. Please state the supplementary item number.

The devices were note certified.

8. Statistics

Number of solar cells tested

☒ Yes ☐ No

A table (and figure) containing information on sample statistics is included in the Supplementary Information.

Explain why this information is not reported/not relevant.

Statistical analysis of the device performance

☒ Yes ☐ No

The mean and standard deviation across multiple devices were calculated and reported in both the main text and the Supplementary Information.

Explain why this information is not reported/not relevant.

9. Long-term stability analysis

Type of analysis, bias conditions and environmental conditions

☐ Yes ☒ No

Provide a description of the type of analysis, bias conditions and environmental conditions (e.g. illumination type, temperature, atmosphere humidity, encapsulation method, preconditioning temperature, bias) for each long-term stability analysis carried out; see ref. 7 and 8 for details.

Not applicable to this work.
